# Novel Copper(II) Complexes Containing β‑Diketones and Imines as Ligands Modulate the Expression of lncRNAs in Triple-Negative Breast Cancer Cells

**DOI:** 10.1021/acsomega.5c06920

**Published:** 2025-12-19

**Authors:** Gislaine Gonçalves Rocha, Luana Munique Sousa Ramos, Laryssa Aparecida Sales Barbosa, Raoni Pais Siqueira, Fernanda Cardoso da Silva, Douglas Cardoso Brandão, Paula Marynella Alves Pereira Lima, André Carlos Pereira de Matos, André Luiz Bogado, Guilherme Pereira Guedes, Jackson Antonio Lamounier Camargo Resende, Gabriele de Menezes Pereira, Pedro Paulo Corbi, Wendell Guerra, Thaise Gonçalves de Araújo

**Affiliations:** † Laboratory of Genetics and Biotechnology, Institute of Biotechnology, 28119 Universidade Federal de Uberlândia, Uberlândia, MG 38700-002, Brazil; ‡ Institute of Chemistry, Universidade Federal de Uberlândia, Av. João Naves de Ávila -2121 - Bloco 1D - Bairro Santa Mônica, Uberlândia, MG CEP 38400-902, Brazil; § Institute of Exact and Natural Sciences of Pontal, Universidade Federal de Uberlândia, Ituiutaba, MG 38304-402, Brazil; ∥ Institute of Chemistry, 28110 Universidade Federal Fluminense, Niterói, RJ 24020-141, Brazil; ⊥ Institute of Exact and Earth Sciences, Universidade Federal de Mato Grosso, Barra do Garças, MT 78600-000, Brazil; # Institute of Chemistry, Universidade Estadual de Campinas, Campinas, SP 13083-970, Brazil; ∇ Laboratory of Nanobiotechnology Prof. Dr. Luiz Ricardo Goulart Filho, Institute of Biotechnoloy, Universidade Federal de Uberlândia, Uberlandia, MG 38405-302, Brazil

## Abstract

In this study, four novel Cu­(II) complexes of the type [Cu­(imine)­(b-diketone)­(NO_3_)], namely, [Cu­(clmp)­(bta)­(NO_3_)]·H_2_O **1**, [Cu­(clmp)­(btacl)­(NO_3_)] **2**, [Cu­(memp)­(bta)­(NO_3_)]·H_2_O **3**, and [Cu­(memp)­(btacl)­(NO_3_)]·H_2_O **4**, in which clmp = 4-chloro-*N*-(pyridin-2-methylene) aniline, memp = 4-methyl-*N*-(pyridin-2-methylene) aniline, bta = (4,4,4-trifluoro-1-phenyl-1,3-butanedionate, and btacl = 1-(4-chlorophenyl)-4,4,4-trifluoro-1,3-butanedionate), were prepared and characterized by elemental analysis, mass spectrometry, conductivity measurements, FT-IR, UV–vis, and single-crystal X-ray diffraction. The spectral and structural data confirmed that the β-diketone anions coordinate to Cu­(II) via the oxygen atoms, while the imine ligands coordinate by the nitrogen atoms. A weakly coordinated nitrate completes the coordination sphere around the metal center. Subsequently, *in vitro* experiments were conducted in MDA-MB231 triple-negative breast cancer cells (TNBC) in which MTT, SRB, LDH, clonogenicity, migration, and caspase activity analyses were performed. lncRNAs associated with epithelial-mesenchymal transition (EMT) were also quantified by qPCR. In the MTT assay, complexes **2** and **3** exhibited IC_50_ values below 20 μM and greater selectivity toward the nontumorigenic MCF-10A cells. The SRB and LDH assays also demonstrated reduced cell viability and increased lactate dehydrogenase release mediated by both complexes. Clonogenicity and migration of TNBC cells were also reduced by **2** and **3**, and an increase in the activity of caspases 3 and 8 was observed, with the most pronounced effects recorded for **3**. Finally, the expression of lncRNAs was downregulated by **2** and **3**, demonstrating the role of the complexes in the modulation of EMT. These findings highlight complexes **2** and **3** as potential antitumor agents for TNBC, emphasizing the importance of exploring the intrinsic mechanisms underlying their anticancer activity.

## Graphical abstract

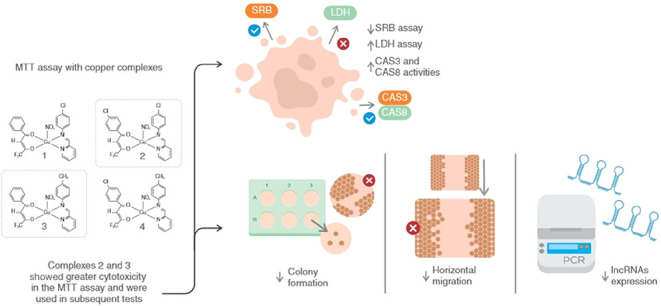

## 1 Introduction

Breast cancer (BC) is the second most common cancer with 2.3 million women worldwide diagnosed in 2022.^1^ In that same year, around 670,000 deaths were recorded, and in the coming years, the incidence and severity of BC is expected to increase due to population aging, lifestyle changes, environmental exposure, and the impacts of the COVID-19 pandemic on access to health systems.^2^

BC is a heterogeneous and complex disease, consisting of multiple histological and molecular subtypes, with different responses to therapy and clinical outcomes.^3,4^ Currently, from a molecular point of view, clinical practice has considered the expression of three main receptors, including estrogen receptor (ER), progesterone receptor (PR), and human epidermal growth factor receptor 2 (HER2). The triple-negative BC (TNBC) subtype does not express any of these markers, represents about 20% of BC cases, and is considered the most aggressive subtype with a high cell proliferation rate, a greater risk of early recurrence, and frequent diagnosis at advanced stages^5,6^

The treatment of TNBC remains a significant therapeutic challenge, with cytotoxic chemotherapy being the main strategy adopted.^7^ This approach plays a fundamental role in both the neoadjuvant and adjuvant settings, aiming to reduce tumor mass and to increase pathological complete response.^8^ However, current drugs are associated with important limitations, such as significant systemic toxicity, development of tumor resistance, and low specificity, which can compromise efficacy and negatively impact patients’ quality of life.^9^ Such scenarios reinforce the urgent need for new anticancer agents,^10^ and copper complexes have emerged as promising potential therapeutic agents.^11^

Copper is an essential micronutrient and a limiting factor in different tumor hallmarks such as proliferation, angiogenesis, and metastasis.^12,13^ In this context, Cu­(I) and Cu­(II) ions coordinated by organic ligands have been explored as a rational anticancer design.^11^ In fact, Cu complexes have shown convenient redox properties and less toxicity compared to platinum complexes.^14^ They can generate reactive oxygen species (ROS), promoting damage to DNA, proteins, and lipids, interfering with replication and transcription.^15,16^ Copper also regulates key signaling pathways, such as MAPK and PI3K/AKT, which are associated with TNBC aggressiveness.^17,18^ Thus, copper complexes can bypass adaptive pathways of tumor resistance and act selectively on highly proliferative cells. Previously, our group demonstrated that Cu­(II) complexes containing long-chain aliphatic hydrazides and 1,10-phenanthroline (1,10-phen) of the type [Cu­(hydrazide)­(1,10-phen)­(H_2_O)]­(NO_3_)_2_ exhibit selective cytotoxicity and inhibit the clonogenicity of TNBC cells MDA-MB-231 by increasing adenosine diphosphate (ADP) hydrolysis and ectonucleoside triphosphate diphosphohydrolase 1 (ENTPD1) transcriptional levels.^19^ Paixão and collaborators demonstrated that the copper complexes [Cu^2+^(4-fluorophenoxyacetic acid hydrazide)­(1,10-phenanthroline)­(ClO_4_)_2_] (**I**) and [Cu^2+^(4-nitrobenzoic hydrazide)­(1,10-phenanthroline)­(ClO_4_)_2_]·H_2_O (**II**) are selective cytotoxic against MDA-MB-231 cells, promoting cell death by apoptosis and interacting directly with DNA. Furthermore, complex **I** induced DNA damage, cell cycle block in G0/G1, and apoptosis mediated by autophagic dysfunction.^20,21^ However, deeper insights into the pertinent and targetable signaling pathways mediated by Cu complexes in TNBC are needed, offering new avenues for therapeutic intervention.

Long noncoding RNAs (lncRNAs) are RNA molecules with more than 200 nucleotides that do not encode proteins and can act as intergenic transcripts, enhancers (eRNAs), or in sense and antisense orientations.^22^ They regulate important cellular processes through interaction with DNA, RNA, and proteins. lncRNAs play crucial roles in tumorigenesis, cell proliferation, metastasis, drug resistance, and programmed cell death in several types of cancer.^23^ Interestingly, lncRNAs, such as MALAT1, HOTAIR, and PVT1 interact with Cu transporters and mitochondrial components, controlling cell survival.^24^ Therefore, noncoding RNAs regulate Cu metabolism and modulate cellular mechanisms, being important targets in predicting the therapeutic response.

In this study, we synthesized and evaluated the *in vitro* effects of four new Cu­(II) complexes on BC cell lines. Furthermore, we investigated the impact of these complexes on the expression of lncRNAs in TNBC cells, highlighting the role of these complexes in the modulation of regulatory molecules of key oncogenic processes.

## 2 Materials and Methods

### 2.1 Chemical Reagents

The organic compounds 4-chloro-*N*-(pyridin-2-methylene) aniline (clmp) and 4-methyl-*N*-(pyridin-2-methylene) aniline (memp) were prepared as described in the literature.^25^ Copper nitrate (Cu­(NO_3_)_2_·3H_2_O) and the ligands Hbta (4,4,4-trifluoro-1-phenyl-1,3-butanedione) and Hbtacl (1-(4-chlorophenyl)-4,4,4-trifluoro-1,3-butanedione) were purchased from Sigma-Aldrich, St. Louis, MO, USA. The structures are presented in Figure 1.

**Figure 1:**
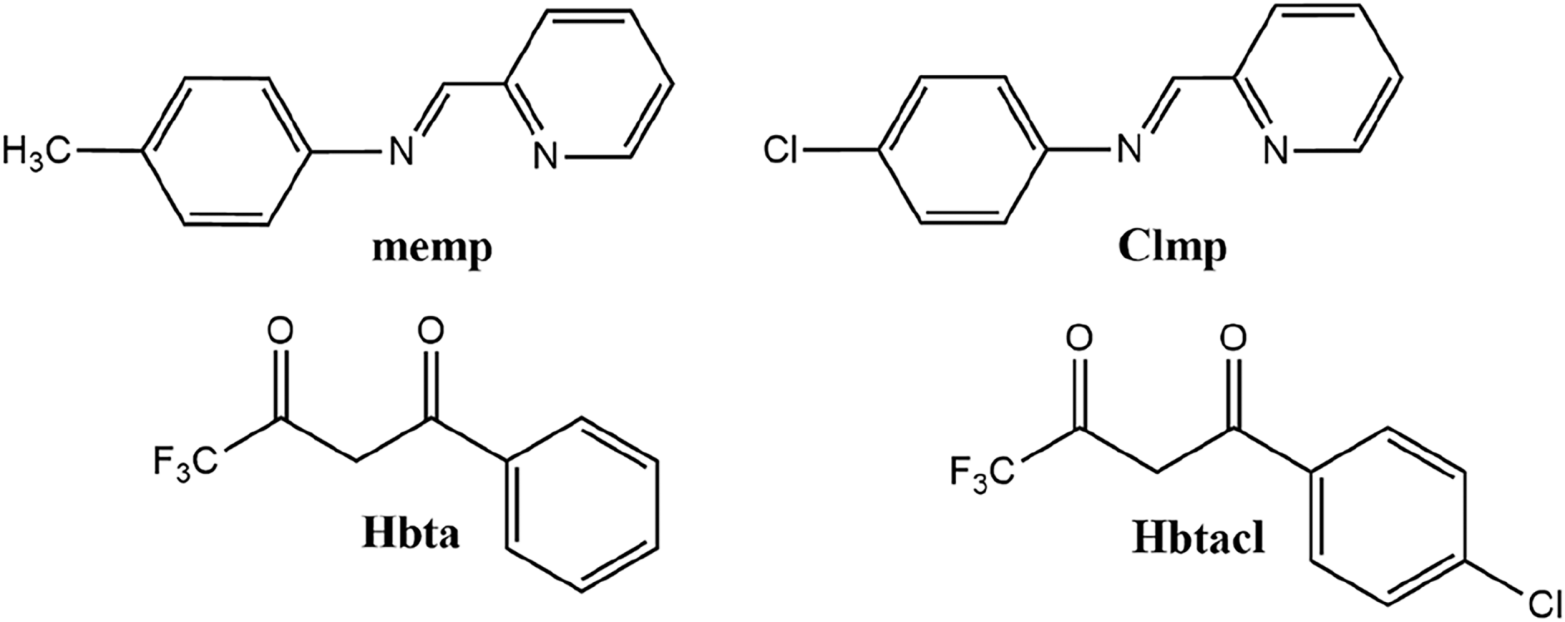
Structure of the ligands used in this work.

#### 2.1.2 Synthesis of Cu­(II) Complexes

The complexes of the type [Cu­(clmp)­(bta)­(NO_3_)]·H_2_O **1**, [Cu­(clmp)­(btacl)­(NO_3_)] **2**, [Cu­(memp)­(bta)­(NO_3_)]·H_2_O **3**, and [Cu­(memp)­(btacl)­(NO_3_)]·H_2_O **4** were prepared according to the following method: in a 25 mL flask, 0.25 mmol of Cu­(NO_3_)_2_·3H_2_O previously solubilized in methanol was added directly to a mixture containing bta or btacl and Clmp or memp, depending on the complex, in a molar ratio of 1:1:1 in methanol. This mixture remained at rest for 15 days until the precipitate formed. The solids produced were then isolated and dried in a desiccator under reduced pressure. Subsequently, they were washed/purified with water/methanol and dried again under reduced pressure. Recrystallization of complex **1** from methanol yielded Cu­(II) complex [Cu­(clmp)­(bta)­(CH_3_OH)]­NO_3_ (see Figure 3), also reported here to confirm the coordination mode of the imine and β-diketone ligands. The CIF file of this structure has been deposited with the Cambridge Crystallographic Data Centre database number 2372590. The supplementary crystallographic data can be obtained, free of charge, via www.ccdc.cam.ac.uk.

##### Complex **1**—[Cu­(clmp)­(bta)­(NO_3_)]·H_2_O

MM (g mol^–1^): 575.38. Yield: 85%. Color: Green. Anal. Calc. for CuC_22_H_17_ClF_3_N_3_O_6_: C, 45.92; H, 2.98; N, 7.30%; Found: C, 45.79; H, 2.72; N, 7.15%. (+)-HRESIMS (methanol), *m*/*z*: 494.0060 [M–NO_3_]^+^ (calc. for CuC_22_H_15_ClF_3_N_2_O_2_, 494.0065 (Δ – 1.01 ppm)). UV–vis (acetonitrile), λmax (ε): 318 (5.47 × 10^4^ M^–1^ cm^–1^), 341 (5.09 × 10^4^ M^–1^ cm^–1^), 645 (4.3 × 10^1^ mol^–1^ L cm^–1^). ΛM (acetonitrile): 105.77 S cm^2^ mol^–1^.

##### Complex **2**—[Cu­(clmp)­(btacl)­(NO_3_)]

MM (g mol^–1^): 591.81. Yield: 60%. Color: Green. Anal. Calc. for CuC_22_H_14_C_l2_F_3_N_3_O_5_: C, 44.65; H, 2.38; N, 7.10%; Found: C, 44.19; H, 2.41; N, 6.97%. (+)-HRESIMS (methanol), *m*/*z*: 529.9650 [M–NO_3_]^+^ (calc. for CuC_22_H_14_Cl_2_F_3_N_2_O_2_, 529.9645 (Δ 0.94 ppm)). ΛM (acetonitrile): 104.21 S cm^2^ mol^–1^.

##### Complex **3**—[Cu­(memp)­(bta)­(NO_3_)]·H_2_O

MM (g mol^–1^): 554.96. Yield: 62%. Color: Green. Anal. Calc. for CuC_23_H_20_F_3_N_3_O_6_: C, 49.78; H, 3.63; N, 7.57%; Found: C, 49.79; H, 3.68; N, 7.27%. (+)-HRESIMS (methanol), *m*/*z*: 474.0610 [M–NO_3_]^+^ (calc. for CuC_23_H_18_F_3_N_2_O_2_, 474.0611­(Δ −0.211 ppm)). ΛM (acetonitrile): 103.85 S cm^2^ mol^–1^.

##### Complex **4**—[Cu­(memp)­(btacl)­(NO_3_)]·H_2_O

MM (g mol^–1^): 589.40. Yield: 77%. Color: Green. Anal. Calc. for CuC_23_H_19_ClF_3_N_3_O_6_: C, 46.87; H, 3.25; N, 7.13%; Found: C, 46.70; H, 3.23; N, 6.74%. UV–vis (acetonitrile), λmax (ε): 337 (4.64 × 10^4^ M^–1^ cm^–1^), 396 (5.82 × 10^2^ M^–1^ cm^–1^), 645 (9.6 × 10^1^ mol^–1^ L cm^–1^). ΛM (acetonitrile): 104.99 S cm^2^ mol^–1^.

#### 2.1.3 Physical Measurements

The percentages of carbon, hydrogen, and nitrogen (CHN) in the samples were determined on a PerkinElmer 2400 elemental analyzer. Conductivity measurements were performed using a Tecnopon mCA-150 conductivity meter using acetonitrile as the solvent. High-resolution mass spectrometry (HRMS) spectra were measured on an Orbi-trap Thermo Q-Exactive (Thermo Fisher Scientific) spectrometer, operating in the positive mode. Samples containing 1.0 mg of the Cu­(II) complexes **1** and **2** were dissolved in 1.00 mL of methanol and then filtered with 22 μm filters and diluted again in the proportion of 20 μL to 1.00 mL of methanol. Methanol:water (1:1) was used as the solvent system, and the samples were infused into the ESI source at a flow rate of 200 μL/min. In the case of complex **3**, a sample containing 1.0 mg of the compound was dissolved in 1.00 mL of methanol and then filtered with a 22 μm filter and diluted again in the proportion of 20 μL to 1.00 mL of methanol. Water:acetonitrile (1:1) with 0.1% v/v HCOOH was used as the solvent system, and the sample was infused into the ESI source at a flow rate of 200 μL/min. The values for the charged complex ions were calculated via the software ChemDraw Ultra 15.0. The UV–vis absorption spectra (acetonitrile, 200–800 nm) were performed on a UV-2501 PC Shimadzu spectrophotometer. Infrared spectra (FTIR) were obtained on a PerkinElmer frontier MIR spectrometer equipped with an attenuated total reflectance (ATR) sample holder with a diamond crystal in the region 4000–400 cm^–1^.

#### 2.1.4 X-ray Diffraction Data

X-ray diffraction data was carried out in Bruker D8 Venture with radiation Mo Kα (λ = 0.71073 Å). The structure was solved by Intrisinc Phasing and refined by full-matrix least-squares on F2 with SHELX package.^26^ The positions of hydrogen atoms were generated geometrically and refined according to a riding model. All non-hydrogen atoms were refined anisotropically. Crystallographic data for the complex [Cu­(clmp)­(bta)­(CH_3_OH)]­NO_3_ – CuC_23_H_19_ClF_3_N_3_O_6_ (MM = 589.40 g/mol): triclinic, space group *P*1̅ (no. 2), *a* = 9.9145(4) Å, *b* = 10.8443(4) Å, *c* = 12.9702(5) Å, α = 72.431(2)°, β = 67.921(2)°, γ = 87.016(2)°, *V* = 1228.98(8) Å^3^, *Z* = 2, *T* = 293 K, μ­(Mo Kα) = 1.065 mm^–1^, *D*_calc_ = 1.593 g/cm^3^, 34584 reflections measured (4.436° ≤ 2Θ ≤ 52.894°), 5052 unique (*R*_int_ = 0.0437, *R*_sigma_ = 0.0251) which were used in all calculations. The final *R*_1_ was 0.0394 (*I* > 2σ­(*I*)), and w*R*_2_ was 0.0913 (all data). Crystallographic data have been deposited with the Cambridge Crystallographic Data Centre database number 2372590.

### 2.2 Cell Culturing

For *in vitro* assays, two BC cell lines were used: MCF7 (ER-positive), cultured in RPMI-1640 medium (Gibco, Thermo Fisher Scientific, Waltham, MA, USA), and MDA-MB-231 (TN), cultured in L15 medium (Gibco, Thermo Fisher Scientific, Waltham, MA, USA). The nontumorigenic mammary cell line MCF10-A was included, cultured in DMEM/F12 medium supplemented with 10 μg/mL of insulin (Gibco, Thermo Fisher Scientific, Waltham, MA, USA), 0.25 μg/mL of hydrocortisone (Sigma-Aldrich, Sigma-Aldrich, St. Louis, MO, USA), and 10 μg/mL of epidermal growth factor (EGF) (Gibco, Thermo Fisher Scientific, Waltham, MA, USA). The cell lines were supplemented with 10% fetal bovine serum (FBS) (Gibco, Thermo Fisher Scientific, Waltham, MA, USA) and 50 μg/mL of gentamicin (Cultilab, Campinas, SP, Brazil); and maintained at 37 °C (Thermo Scientific Forma Series 3 Water Jacketed CO_2_ Incubator, Waltham, MA, USA). Only the MDA-MB-231 cell line was cultured without CO_2_ gas exchange. The three lineages were obtained from the American Type Culture Collection (ATCC; Manassas, VA, USA) and confirmed as free from mycoplasma contamination.

### 2.3 MTT Assay

The cytotoxic effects of complexes **1–4** were evaluated using MTT reagent (3-[4,5-dimethylthiazol-2-yl]-2,5-diphenyltetrazolium bromide, Invitrogen, Waltham, MA, USA). The initial screening was performed only on BC cell lines, and the selectivity was assessed only for the compounds that reduced BC cells viability by 50%. The cell lines MCF10A (1.2 × 10^4^ cells/well), MCF7 (5.0 × 10^3^ cells/well), and MDA-MB-231 (1.5 × 10^4^ cells/well) were seeded into 96-well plates. The Cu­(II) compounds were resuspended in 0.5% dimethyl sulfoxide (DMSO, Sigma-Aldrich, St. Louis, MO, USA) and serially diluted in appropriate culture medium (in a range of 1.25–160 μM). Treatments were carried out for 24 and 48 h, and after this period, the MTT reagent (0.5 mg/mL) was added and incubated for 4 h at 37 °C, when 200 μL of DMSO was used to solubilize the formazan crystals. Finally, the absorbance was measured at 560 nm using a Multiskan FC Microplate Reader FC (Thermo Fisher Scientific). Untreated cells were included as a negative control and considered as 100% of cell viability. Cytotoxicity was calculated using the following formula: Cytotoxicity (%) = [(absorbance of cells treated with the compounds/absorbance of cells treated with DMSO) × 100]. A dose–effect graph was plotted with the logarithm of Cu­(II) complexes concentration as the *x*-axis and cell viability percentage as the *y*-axis to obtain the IC_50_ value. The Selectivity Index (SI) was calculated as the ratio between the IC_50_ of the nontumorigenic cell line (MCF-10A) and the IC_50_ of the BC cells, with values greater than 2.0 considered to be significant.^27^

### 2.4 Sulforhodamine B (SRB) and Lactate Dehydrogenase (LDH) Released

The TNBC MDA-MB-231 cell line and complexes **2** and **3** were chosen for subsequent experiments due to the cytotoxicity and selectivity observed in the MTT assay. The cells were cultured as described above and seeded in 96-well plates (1.5 × 10^4^ cells/well). For the SRB assay, TNBC cells were treated with serial concentrations starting from twice the previously calculated IC_50_ for complex **2** (1.84 to 29.5 μM) and complex **3** (2.06 to 33 μM). After 48 h, cells were fixed with 50% (w/v) trichloroacetic acid (TCA) and stained with 0.04% (w/v) SRB solution (Invitrogen, Waltham, MA, USA). The plates were then washed repeatedly, and the dye was solubilized with an alkaline 10 mM Tris-HCl solution (pH 10.5). Absorbance was measured with a Multiskan FC spectrophotometer (Thermo Fisher Scientific, Waltham, MA, USA) at a wavelength of 510 nm. The LDH assay was performed using the LDH kit (Sigma-Aldrich, St. Louis, MO, USA) following the manufacturer’s instructions. MDA-MB-231 cells were treated for 48 h with 7.37 μM (IC_25_) and 14.75 μM (IC_50_) of complex **2**; and 8.25 μM (IC_25_) and 16.50 μM (IC_50_) of complex **3**. Absorbances were measured at 492 and 620 nm using a Multiskan FC microplate reader (Thermo Fisher Scientific, Waltham, MA, USA). For maximum control of LDH release (positive control), three wells containing only the substrate and enzyme were included. Doxorubicin (IC_50_ = 0.42 μM; IC_25_ = 0.21 μM) and Docetaxel (IC_50_ = 20.7 μM; IC_25_ = 10.35 μM) (Sigma-Aldrich, St. Louis, MO, USA) were included for comparison, since they are well-characterized drugs that induce LDH release. The IC_50_ values were used as previously described.^19^

### 2.5 Colony Formation

MDA-MB-231 cells were seeded into 6-well plates (5 × 10^2^ cells/well) and treated with 7.37 μM (IC_25_) and 14.75 μM (IC_50_) of complex **2**; and 8.25 μM (IC_25_) and 16.50 μM (IC_50_) of complex **3**. After 48 h, the treatments were removed, and complete medium was added to the wells. This procedure was repeated every 3 days. After 14 days, the cells were washed with 1× phosphate-buffered saline (PBS), fixed with formaldehyde (4% v/v), and visualized with crystal violet solution (0.5% v/v). Photographs of the colonies were captured using iBright Imaging Systems (Thermo Fisher Scientific, Waltham, MA, USA). For quantitative analysis, 33% m/v acetic acid was added to the wells and absorbance readings were taken at a wavelength of 570 nm using the Multiskan FC microplate reader (Thermo Fisher Scientific, Waltham, MA, USA).

### 2.7 Horizontal Migration

Horizontal migration was assessed by the wound healing method.^28^ Briefly, MDA-MB-231 cells were seeded into 12-well plates (2 × 10^5^ cells/well), and once confluence was reached, scratches were made on the surface of the monolayer using 200 μL pipet tips. After washing with PBS 1×, culture medium containing 3.69 μM (IC_12.5_) and 7.37 μM (IC_25_) of complex **2** and 4.125 μM (IC_12.5_) and 8.25 μM (IC_25_) of complex **3** was added. The choice of these concentrations aimed to evaluate the effects of the compounds on cell migration at levels that did not significantly compromise cell viability.

Representative images were captured at time intervals of 0, 24, and 48 h. Untreated cells were included as a negative control. Analysis was performed using ImageJ v. 1.54g software, and the wound closure was measured as a percentage using the following formula: (Initial Area – Final Area)/Initial Area × 100.

### 2.8 Caspases 3 and 8 Activities

To investigate the proapoptotic effect of complexes **2** and **3**, the activities of caspase 3 (CAS3) and caspase 8 (CAS8) enzymes were measured colorimetrically. MDA-MB-231 cells were seeded at a density of 5.0 × 10^6^ cells per well and treated for 48 h with 7.37 μM (IC_25_) of complex **2** and 8.25 μM (IC_25_) of complex **3**. Protein extraction was performed using the NE-PER kit (Thermo Fisher Scientific, St. Louis, MO, USA), according to the manufacturer’s instructions. Then, caspase activity assays were performed in a 96-well plate, in which 10 μL of the selective substrate for caspase 3 (*N*-acetyl-Asp-Glu-Val-Asp *p*-nitroanilide, Sigma-Aldrich, St. Louis, MO, USA) or caspase 8 (*N*-acetyl-Ile-Glu-Thr-Asp *p*-nitroanilide, Sigma-Aldrich, St. Louis, MO, USA) was added to a final volume of 100 μL in assay buffer in each well.

### 2.9 lncRNAs Quantification

MDA-MB-231 cells were seeded at a density of 1 × 10^5^ cells per well and treated for 48 h with 3.69 μM (IC_12.5_) of complex **2** and 4.125 μM (IC_12.5_) of complex **3**. Total RNA was extracted using Trizol reagent (Invitrogen, Waltham, MA, USA) as indicated by the supplier. RNA quality and quantity were assessed using a Nanodrop 1000 instrument (Thermo Fisher, Waltham, MA, USA). For reverse transcription, the M-MLV Reverse Transcriptase Kit (Invitrogen, Waltham, MA, USA) was used, and procedures were performed according to the manufacturer’s instructions. qPCR reactions were conducted using the StepOnePlus system (Applied Biosystems, Waltham, MA, USA), with a Power SYBR Green PCR Master Mix (Applied Biosystems, Waltham, MA, USA). Melting curves were monitored, and quantification was determined through normalization with the reference gene β-2-microglobulin (*β2M*). The comparative Cq method was applied to assess the relative expression of the following genes: Long noncoding RNA activated by TGF-β (*lncATB*), Long intergenic nonprotein coding RNA 958 (*lncLINC00958*), Bladder cancer-associated transcript 2 (*lncBLACAT2*), Long intergenic nonprotein coding RNA 941 (*lncLINC00941*), Long intergenic nonprotein coding RNA 1278 (*lncLINC01278*), MAGI1 Intronic Transcript 1 (*lncMAGI1-IT1*), MIR22 host gene (*lncMIR22HG*), OIP5 antisense RNA 1 (*lncOIP5-AS1*), and ZNFX1 antisense RNA 1 (*lncZFAS1*). *β2M* was used as the reference gene.^29^ The sequences of the primers for lncRNAs targets are detailed in Table 1.

**Table 1:** Primers Sequences Used for lncRNAs Quantification

lncRNA	Primer sequence (Forward – Reverse) 5′ – 3′	Amplicon (pb)	Reference
*ATB*	F: TCTGGCTGAGGCTGGTTGAC	142	^30^
	R: ATCTCTGGGTGCTGGTGAAGG		
*BANCR*	F: ACAGGACTCCATGGCAAACG	80	^31^
	R: ATGAAGAAAGCCTGGTGCAGT		
*LINC00958*	F: AGATAGCTCCAGGTTGGATT	102	^32^
	R: GGCGTCTGTGTAGTGTTCA		
*LINC00941*	F: CCTCCAACCCCCTTTTCTCC	140	^33^
	R: GAAGGCAGGAAGTCTGTGCT		
*LINC01278*	F: CCTGGTGTGCTGGCATCAAGTA	136	^34^
	R: TCTCCACTTCGCCACGGTCT		
*MAGI1-IT1*	F: TGATGCTGCTGATCTGGTCT	122	^35^
	R: GCCAAGTCTCTGCTCGTACC		
*MIR22HG*	F: CCATACATTGCGTGTGGGAG	136	^36^
	R: TTCGTAGGTCAAATGACATGGAG		
*OIP5-AS1*	F: TGCGAAGATGGCGGAGTAAG	136	^37^
	R: TAGTTCCTCTCCTCTGGCCG		
*ZFAS1*	F: ACGTGCAGACATCTACAACCT	97	^38^
	R: TACTTCCAACACCCGCAT		

### 3.0 Statistical Analysis

The results were analyzed using GraphPad Prism 9.0 software (GraphPad Software Inc., La Jolla, CA, USA). After confirming the normal distribution of the data, the ANOVA test was performed, followed by Tukey’s post hoc test for multiple comparisons. The data are presented as the mean ± standard deviation. Statistically significant differences were considered when *p* < 0.05.

## 4 Results and Discussion

### 4.1 Chemistry

Four new Cu­(II) complexes containing β-diketone (4,4,4-trifluoro-1-phenyl-1,3-butanedione—Hbta or 1-(4-chlorophenyl)-4,4,4-trifluoro-1,3-butanedione—Hbtacl) and imine (4-chloro-*N*-(pyridin-2-methylene) aniline—clmp or 4-methyl-*N*-(pyridin-2-methylene) aniline—memp) as ligands were prepared and characterized. The complexes are similar to other copper complexes previously reported by our research group.^39–41^ The only difference consists of the presence of an imine ligand instead of 1,10-phenanthroline or 2,2-bipyridine. As to the characterization of these complexes, the results of the elemental analyses are in accordance with the proposed structures (Figure 2) and the molar conductivity values (ΛM ≈ 105 S cm^2^ mol^–1^) for these Cu­(II) complexes (10^–3^ M; acetonitrile) fall in the range observed for 1:1 electrolytes,^42^ which indicates the labilization of the axial ligand (nitrate anion) in solution. The high-resolution mass spectra are also in agreement with the proposed structures (Experimental Section and Figures S9–S11). For instance, the mass spectrum of complex **3** exhibited the charged ion at *m*/*z* 474.0610 [M–NO_3_]^+^ (calc. for CuC_23_H_18_F_3_N_2_O_2_, 474.0611 (Δ 0.21 ppm)). As to the UV–vis spectra (Supporting Information), these complexes exhibited a broad and asymmetric d-d band centered at ≈645 nm, which is in agreement with the results found for similar complexes with square pyramidal geometry reported by our research group.^39^ The complex **1**, for example, exhibited a d-d band (Figure S2) centered at 645 nm (ε = 43 mol^–1^ Lcm^–1^). In the IR spectra of metal complexes, absorption bands between 3082 and 3011 cm^–1^ can be assigned to the ν_CH_ and a band close to 1600 cm^–1^ is due to the presence of the C = O group. For all complexes, three bands around 1400 (ν_a_NO_2_), 1295 (ν_s_NO_2_), and 950 cm^–1^ (νNO) suggest the presence of a unidentate nitrate group, as expected for its C_2 V_ symmetry. Furthermore, the separation values of the two highest frequency bands (ν_a_NO_2_ – νsNO_2_ = ≈100 cm^–1^) corroborate with a nitrate group coordinated in a monodentate manner through the oxygen atom.^43^ As to the CF_3_ group, the symmetric and asymmetric stretching frequencies can be seen around 1138 and 1320 cm^–1^, respectively, and for complexes **1**, **3**, and **4**, a broad band close to 3400 cm^–1^ suggests the presence of a water molecule.

**Figure 2:**
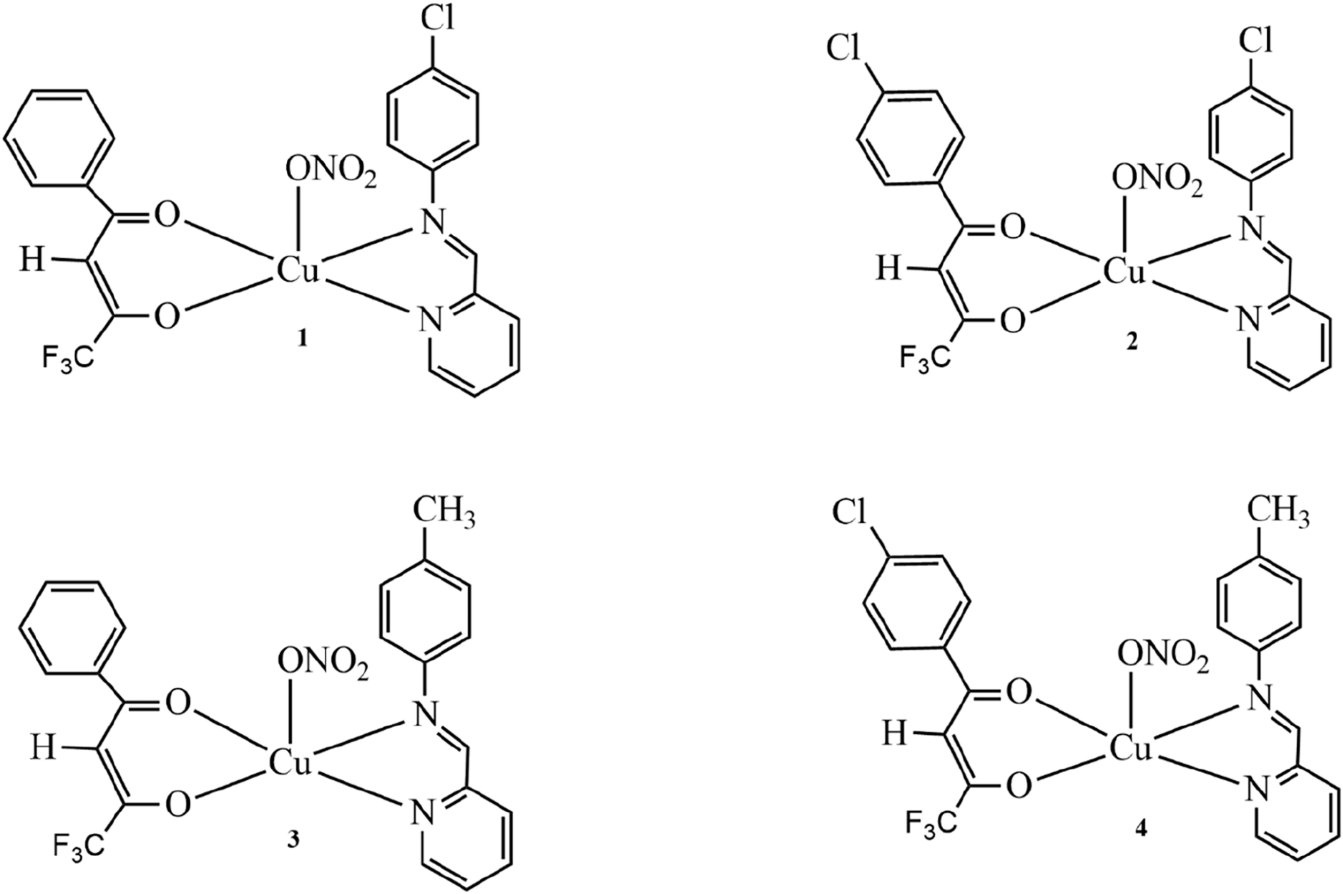
Proposed structures for the Cu­(II) complexes. [Cu­(clmp)­(bta)­(NO_3_)]·H_2_O **1**, [Cu­(clmp)­(btacl)­(NO_3_)] **2**, [Cu­(memp)­(bta)­(NO_3_)]·H_2_O **3**, and [Cu­(memp)­(btacl)­(NO_3_)]·H_2_O **4**.

Finally, recrystallization of complex **1** from methanol yielded crystals of the general formula [Cu­(clmp)­(bta)­(CH_3_OH)]­NO_3_, the structure of which is briefly described here. The crystal data show that the complex [Cu­(clmp)­(bta)­(CH_3_OH)]­NO_3_ crystallized as a nitrate salt, with the copper atom exhibiting a square pyramidal coordination sphere (Figure 3). A weakly coordinated methanol molecule, which can be readily dissociated from the metal in solution, is observed at the apical position with a longer Cu­(II)–O bond distance (2.225(2) Å) compared to the basal Cu–N/O values (1.9241 to 2.0146 Å). The longer Cu–O3 bond length can be explained by the Jahn–Teller effect, as reported for similar Cu­(II) complexes with distorted square pyramidal geometry,^39,40^ including those with methanol coordinated in the apical position.^44–46^ Methanol molecules play a role in the crystal packing of the compound, connecting the complex cation to the nitrate anion via H-bond. As expected, the crystal data also showed that the β-diketones coordinated to the metal via the oxygen atoms, while the pyridine-imine ligand coordinates via its two nitrogen atoms. The selection of bond lengths and bond angles is presented in Table 2.

**Figure 3:**
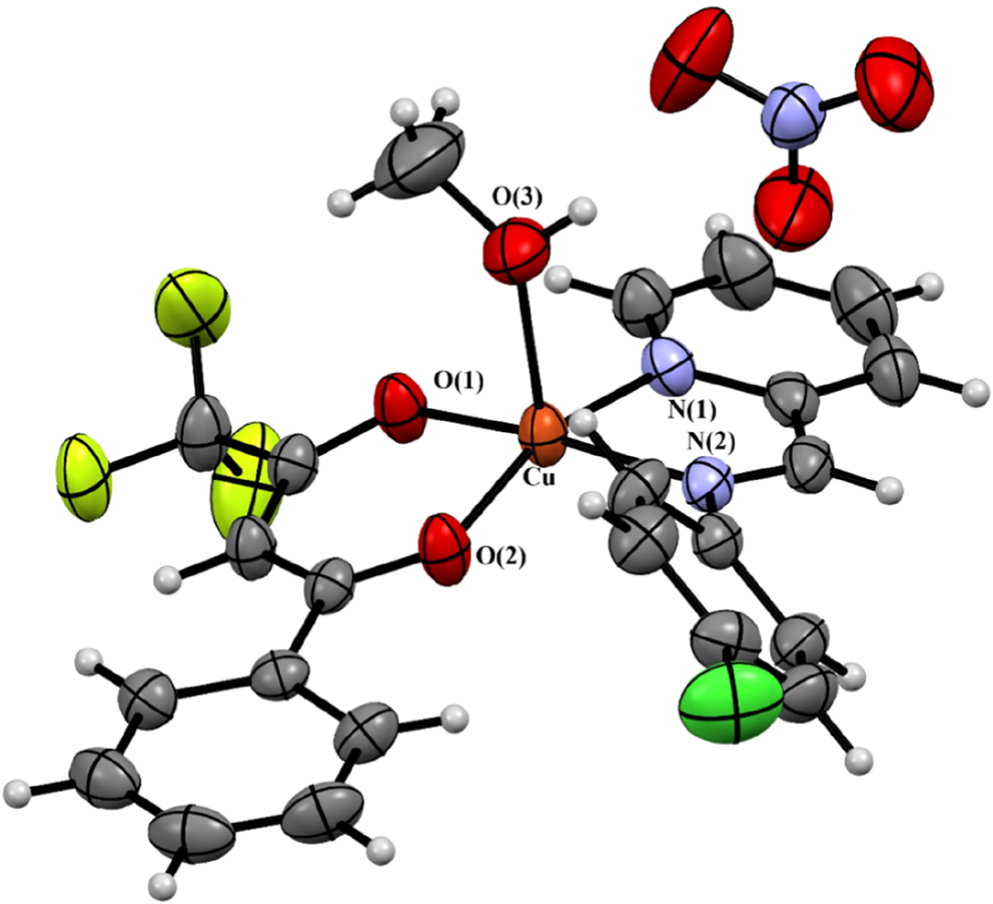
X-ray crystal structure of the complex [Cu­(clmp)­(bta)­(CH_3_OH)]­NO_3_ isolated from the recrystallization of complex **1** in methanol.

**Table 2:** Selected Bond Lengths [Å] and Angles [°] for the Complex [Cu­(clmp)­(bta)­(CH_3_OH)]­NO_3_

Bond lenghts	
Cu–O(2)	1.9294(17)
Cu–O(1)	1.9241(17)
Cu–N(2)	2.0146(19)
Cu–N(1)	1.993(2)
Cu–O(3)	2.225(2)

Bond angles	
O(2)–Cu-N(2)	91.19(8)
O(2)–Cu-N(1)	162.30(8)
O(2)–Cu-O(3)	99.67(9)
O(1)–Cu-O(2)	92.43(7)
O(1)–Cu-N(2)	167.85(8)
O(1)–Cu-N(1)	91.80(8)
O(1)–Cu-O(3)	95.75(8)
N(2)–Cu-O(3)	95.07(8)
N(1)–Cu-N(2)	81.40(8)
N(1)–Cu-O(3)	97.00(9)

### 4.2 Cytotoxicity of the Cu­(II) Complexes

Initially, an MTT assay was performed on BC cells, and Table 3 presents the IC_50_ values. BC is currently the most diagnosed type of cancer among women worldwide, and, despite significant advances in molecular diagnosis, surgical interventions, and chemotherapy, patient survival is still impacted by resistance and toxicity, mainly in cases of aggressive tumors.^47^ Therefore, therapeutic failure, early recurrence, and increased metastatic potential challenges TNBC management^7^ and highlights the need of new compounds and innovative strategies capable of circumventing this scenario.

**Table 3:** Half-Maximal Inhibitory Concentration (IC_50_) Values for Cu­(II) Complexes against MCF7 and MDA-MB-231 Breast Cancer Cells after 24 and 48 h of Treatment

IC_50_ (μM)				
	24 h		48 h	
Complex	MCF7	MDA-MB-231	MCF7	MDA-MB-231
**1**	>160	34.52	>160	35.33
**2**	>160	22.78	>160	14.75
**3**	>160	39.25	>160	16.50
**4**	>160	81.25	62.87	19.00

The four new compounds synthesized here did not reduce the viability of the MCF-7 cell line by more than 50% after 24 or 48 h. Thus, the IC_50_ value was calculated only for complex **4**, but it was higher than 50 μM. For the TNBC cell line MDA-MB-231, all complexes showed a time- and dose-dependent response profile, with IC_50_ values lower than 50 μM after 48 h of treatment. The lower IC_50_ values were observed for complexes **2** and **3**, indicating a greater cytotoxicity of these complexes against the TNBC cell line used in this study.

As the complexes presented IC_50_ < 50 μM in the MDA-MB-231 cell line, their activity in the nontumor cell line MCF-10A was then evaluated to determine the selectivity index (SI) (Figure 4). Complex **1** did not show SI < 2.0 at any of the evaluated time points. Complexes **2**, **3**, and **4** demonstrated the highest selectivity over 48 h treatment, with greater activity over time in the TNBC cell line, since an increase in SI and a decrease in IC_50_ were observed in MDA-MB-231 cells. Although complex **4** presented an IC_50_ close to 20 μM, only complexes **2** and **3** were selected for further experiments, since compounds with IC_50_ lower than 20 μM are often considered promising in anticancer studies.^48^

**Figure 4:**
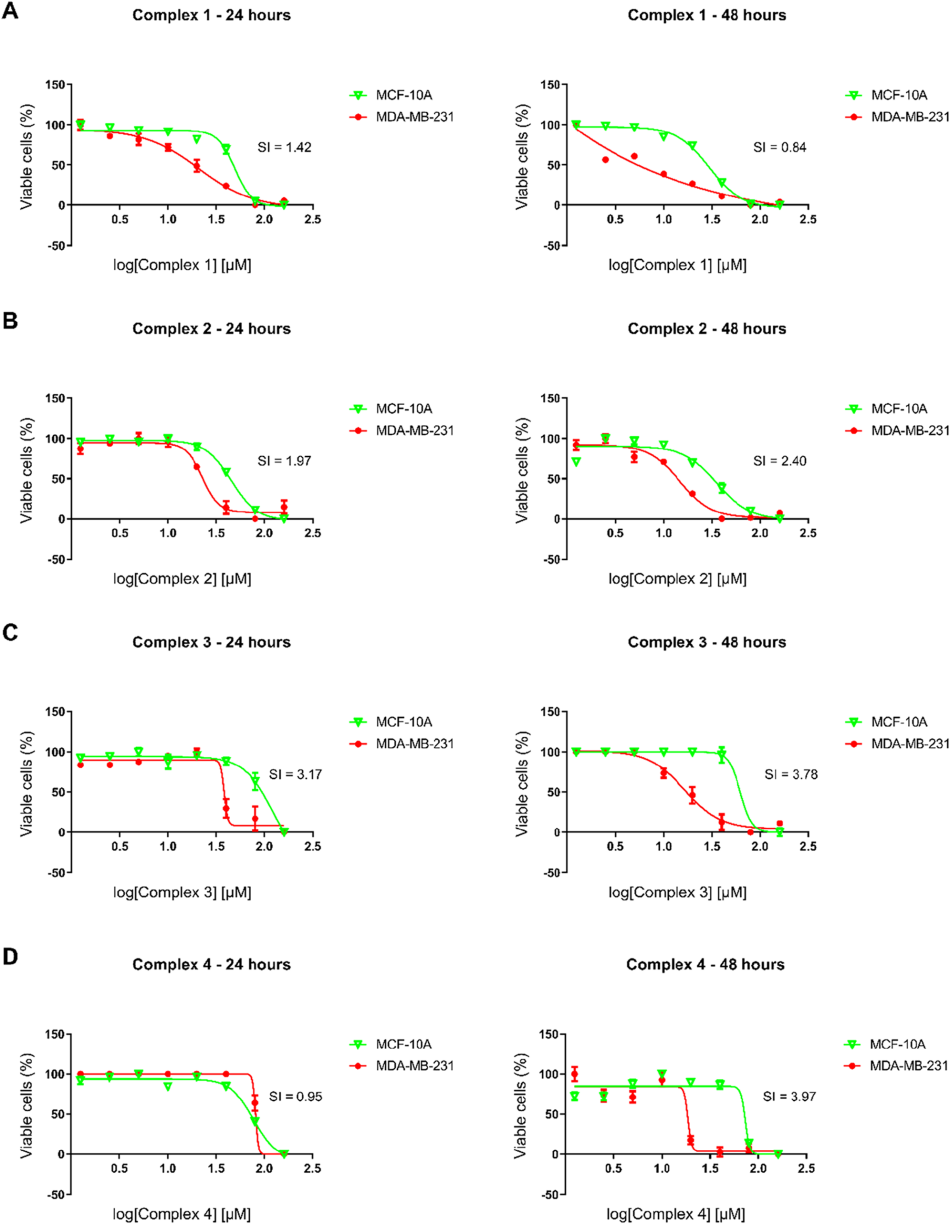
Effect of complexes **1**, **2**, **3**, and **4** on the viability of MCF-10A breast cells (nontumorigenic) and MDA-MB-231 cells (triple-negative breast cancer). Treatments were performed for 24 and 48 h with increasing concentrations (1.25–160 μM) of the complexes. (A) Complex **1**. (B) Complex **2**. (C) Complex **3**. (D) Complex **4**. Cell viability was determined using an MTT assay. Results are expressed as the mean ± standard deviation. Selectivity index (SI) was calculated by dividing the IC_50_ value for nontumor cell lines, MCF10A, by the IC_50_ values for MDA-MB-231.

Finally, the cytotoxicity of the ligands btacl, bta, clmp, and memp were evaluated against the MCF-10A and MDA-MB-231 cell line following 48 h of treatment (Supporting Information). None of the ligands exhibited cytotoxic activity, suggesting that the observed effects are attributable to the Cu­(II) complexes rather than the individual ligands.

To confirm the MTT results, the SRB assay, which quantifies the total cellular protein content, and the LDH assay, which evaluates the release of lactate dehydrogenase into the extracellular medium, was performed on the TNBC cell line (Figure 5). For the SRB assay, concentrations of 1.84 to 29.5 μM were used for complex **2** and 2.06 to 33 μM for complex **3**, established from twice the IC_50_ previously obtained in the MTT. In the LDH assay, the concentrations corresponding to the IC_25_ and IC_50_ of each compound (7.37 and 14.75 μM for complex **2**; 8.25 and 16.50 μM for complex **3**) were used. The results for SRB showed a dose–response curve pattern for both complexes, confirming the data obtained from the MTT assay (Figure 5A,B). The release of the LDH enzyme into the extracellular medium increased in a concentration-dependent manner for **2** and **3**, suggesting damage to the integrity of the plasma membrane (Figure 5C,D). The release of LDH induced by complexes **2** and **3** did not differ significantly from that of Doxorubicin and Docetaxel. It is important to highlight that both doxorubicin and docetaxel are not selective drugs, as already demonstrated in a previous publication.^19^ These data reinforce the findings of the MTT assay, indicating the cytotoxic effect of complexes **2** and **3** on the MDA-MB-231 cell line.

**Figure 5:**
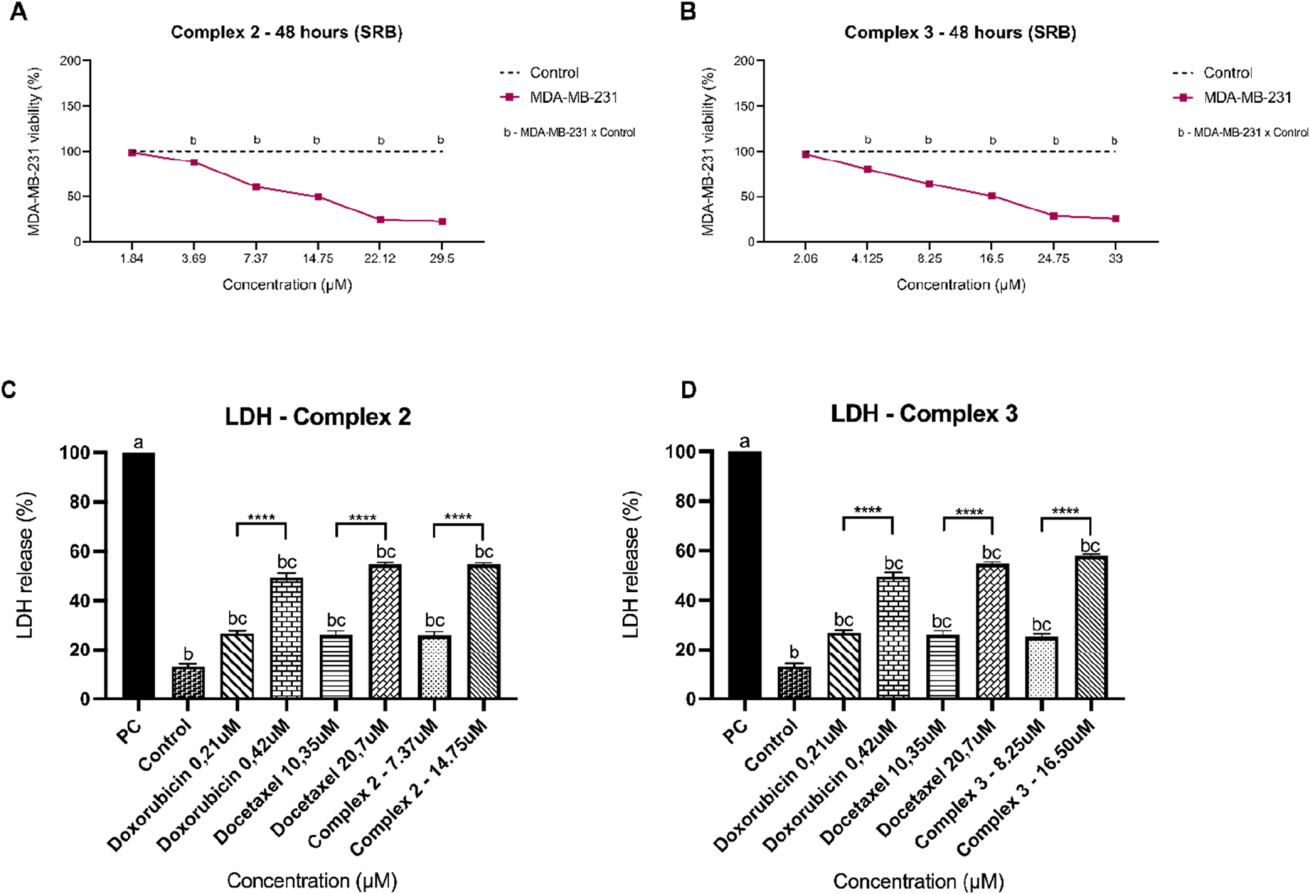
Sulforhodamine B (SRB) and lactate dehydrogenase (LDH) release from the MDA-MB-231 cell line after treatment with complexes **2** and **3**. (A) Treatment for 48 h with complex **2**. (B) Treatment for 48 h with complex **3**. The control refers to untreated cells. Treatments identified with the letter b differed from the control statistically calculated using ANOVA followed by Tukey’s post hoc test. (C) Percentage of LDH release from treatments with Complex **2**. (D) Percentage of LDH release from treatments with complex **3**. Control: untreated cells. PC: positive control, maximum LDH release. Treatments identified with the letter b differed statistically from the positive control, with letter c differed from untreated cells, and *****p < 0.00001. Doxorubicin and Docetaxel were included for comparison. Statistical significance was also calculated using ANOVA followed by Tukey’s post hoc test.

### 4.3 Effects on Clonogenicity and Cell Migration

The colony formation assay was used to evaluate the long-term proliferative potential of the MDA-MB-231 cell line, reflecting the ability of individual cells to survive, divide, and originate colonies after exposure to the treatments. This assay is especially relevant for aggressive tumor cells, such as those of the MDA-MB-231 lineage, which have high clonogenic capacity and are associated with greater metastatic potential.^49^ To perform the experiment, concentrations corresponding to the IC_50_ and IC_25_ of each compound were used. The results, presented in Figure 6, reveal that both complexes reduced the clonogenic capacity of MDA-MB-231 cells (Figure 6A,B). In addition, the impact of treatments on the TNBC cell line was also investigated through a wound healing assay. The complexes significantly inhibited cell migration when applied at concentrations corresponding to IC_25_ (Figure 6C,D). These findings reinforce the efficacy of complexes **2** and **3** in suppressing clonogenicity and migration in TNBC cells, processes that are critical for tumor progression and metastasis.

**Figure 6:**
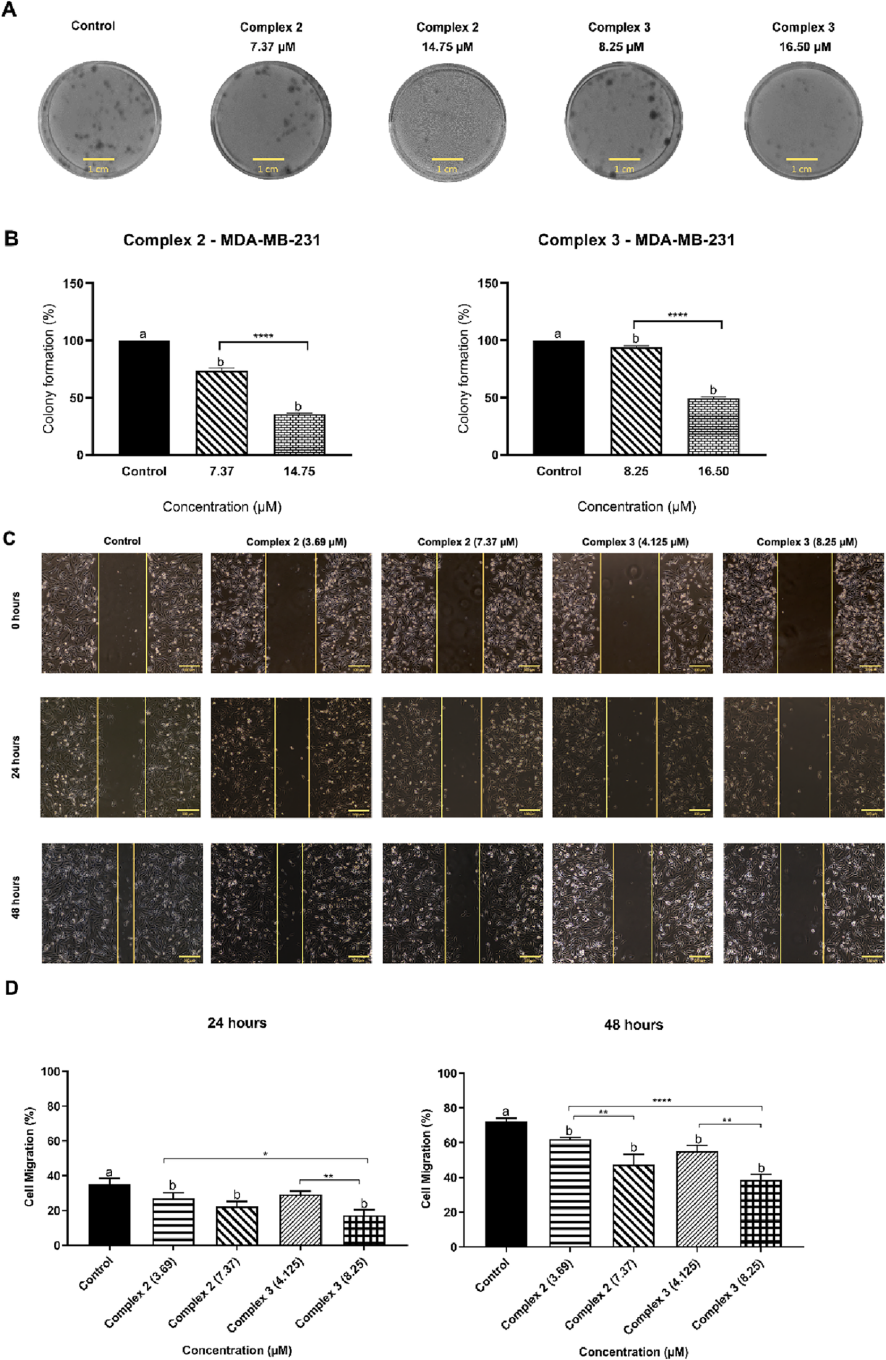
Effects of complexes **2** and **3** on clonogenicity and migration of triple-negative breast cancer cells. (A) Representative images of wells and colonies formed by MDA-MB-231 lineage. (B) Percentage of MDA-MB-231 colonies formed following 48 h of treatment with **2** and **3**. (C) Representative images of cell migration. (D) Quantification of MDA-MB-231 cell migration after treatment with **2** and **3** for 0, 24, and 48 h. Experiments were performed in triplicate, and results are presented as the mean ± standard deviation. Statistical significance was determined using ANOVA followed by Tukey’s post hoc test (*p < 0.05; **p < 0.01; ***p < 0.0001; ****p < 0.00001). Treatments identified with the letter *b* differed from the control statistically.

### 4.4 Enzymatic Activity of CAS3 and CAS8

The enzymatic activity of caspases CAS3 and CAS8 was also analyzed in the MDA-MB-231 cell line after treatment with complexes **2** and **3**, using concentrations corresponding to the IC_25_ of each compound. It was observed that the treatments led to an increase in the expression of both caspases, indicating the activation of the apoptotic mechanism (Figure 7). Furthermore, the results showed that the caspase activities in cells treated with complex **3** were more pronounced compared to those treated with complex **2**. Thus, treatment with both complexes activates both the early signals of apoptosis and its final phase.

**Figure 7:**
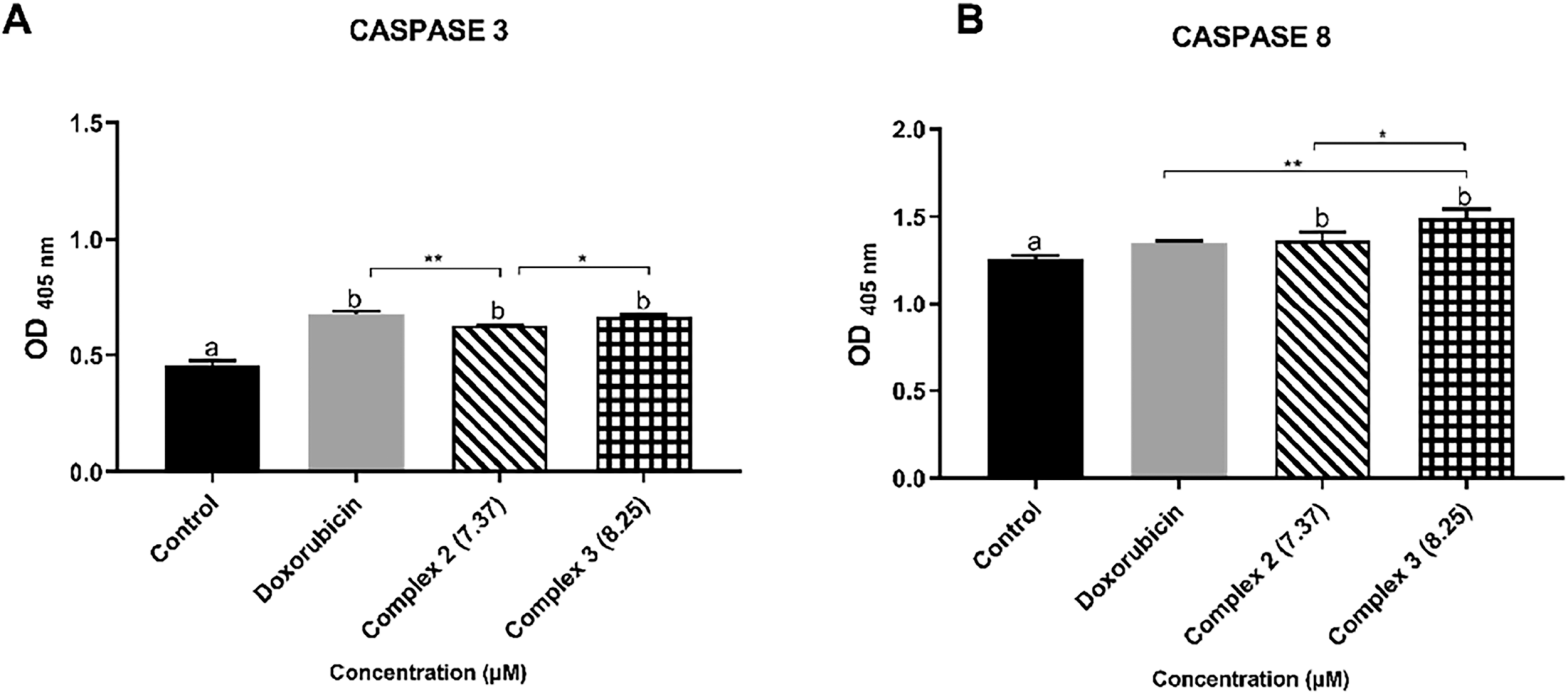
Effect of complexes **2** and **3** on caspase activation in the MDA-MB-231 cells. (A) Caspase 3 activity and (B) Caspase 8 activity. Cells were treated with the IC_25_ of the complexes for 48 h. Quantification was performed by measuring optical density (OD) at 405 nm. Results are expressed as mean ± standard deviation. Statistical significance was calculated using ANOVA followed by Tukey’s post hoc test (*p < 0.05; **p < 0.01; ***p < 0.0001; ****p < 0.00001). Treatments identified with the letter b differed from the control statistically.

### 4.5 Transcriptional Modulation Mediated by Treatment with Complexes **2** and **3**

Finally, to investigate the potential modulatory effects of complexes **2** and **3** on noncoding transcripts associated with tumor progression, the transcriptional levels of *lncATB*, *lncBANCR*, *lncBLACAT2*, *lncLINC00941*, *lncLINC01278*, *lncMAGI1-IT1*, *lncMIR22HG*, *lncOIP5-AS1*, and *lncZFAS1* were quantified in MDA-MB-231 cells (Figure 8). Treatment with 3.69 μM (IC_12.5_) of complex **2** resulted in a significant decrease in the level of expression of the genes *lncATB, lncBANCR, lncBLACAT2, lncLINC00941*, and *lncOIP5-AS1*. Additionally, treatment with 4.125 μM (IC_12.5_) complex **3** also reduced the expression of transcripts for *lncBANCR, lncBLACAT2, lncLINC00941, and lncOIP5-AS1*. For the other transcripts evaluated, no statistically significant changes were observed. lncRNAs signatures have been investigated with prognostic potential, being associated with different cellular death mechanisms, including necroptosis in BC,^50^ ferroptosis in hepatocellular carcinoma^51^ and cuproptosis in lung cancer.^52^ However, the role of lncRNAs linked to Cu-induced anticancer mechanisms is poorly understood and is still under investigation. In this sense, our study is pioneering, revealing that Cu­(II) complexes change the expression profile of lncRNAs related to the epithelial-mesenchymal transition (EMT), which is a key event for the aggressiveness of TNBC cells.

**Figure 8:**
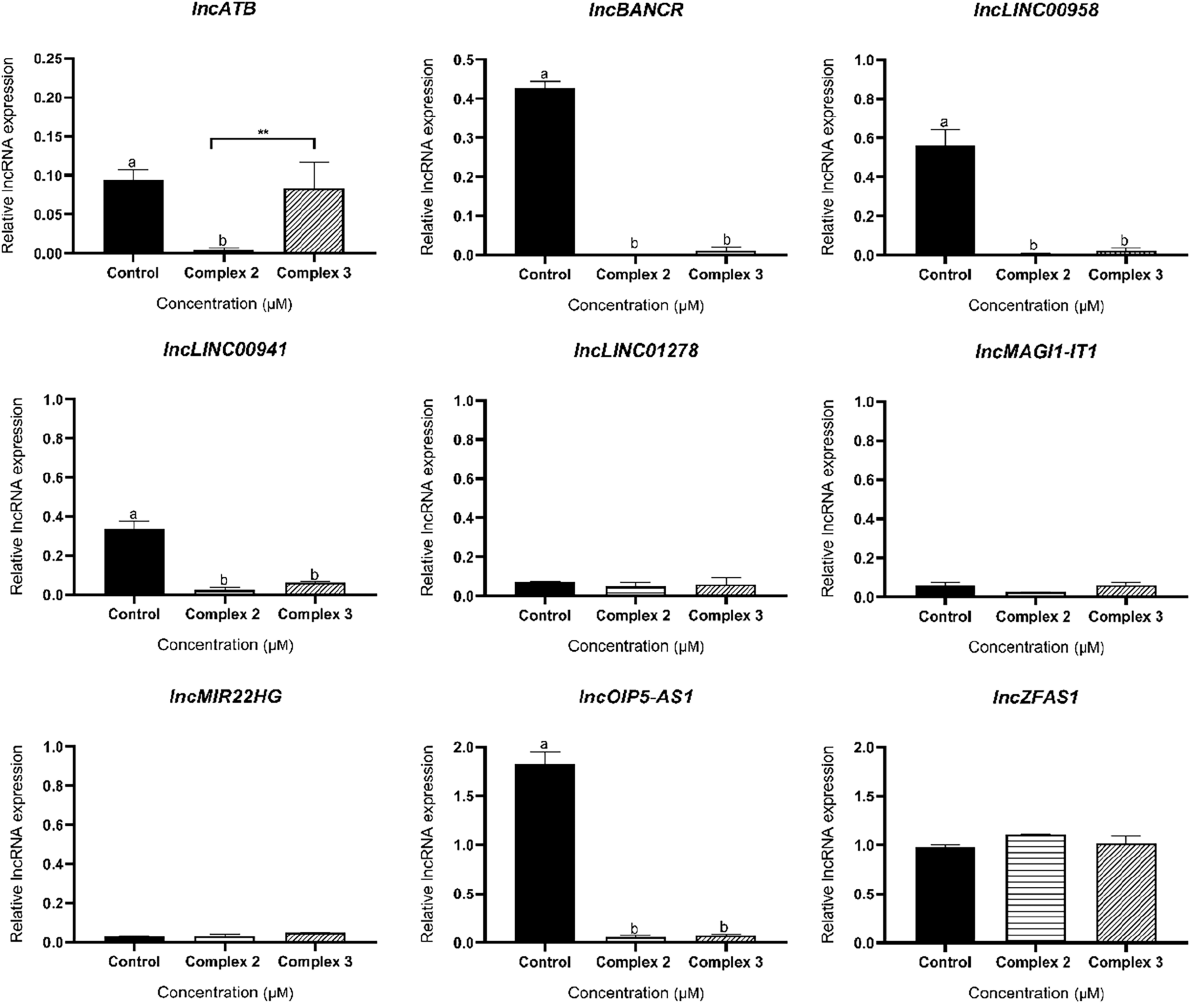
Relative expression levels of *lncATB, lncBANCR, lncBLACAT2, lncLINC00941, lncLINC01278, lncMAGI1-IT1, lncMIR22HG, lncOIP5-AS1*, *and lncZFAS1* after treatment of MDA-MB231 (triple-negative breast cancer) cells with IC_12.5_ values of complexes **2** and **3** for 48 h. Results are expressed as mean ± standard deviation. Statistical significance was determined using Student’s *t* test (*p < 0.05; **p < 0.01; ***p < 0.0001; ****p < 0.00001). Treatments identified with the letter *b* differed from the control statistically.

*lncATB* has been extensively described as a key regulator of tumor progression and the acquisition of metastatic phenotypes. *lncBANCR* is a promoter of cell migration, and its overexpression in BC is associated with a worse prognosis, favoring proliferation, colony formation, invasion, and metastasis.^37,53^ *lncLINC00958* is widely recognized as a positive regulator of tumor progression, involved in cell proliferation, apoptosis evasion and resistance to therapies.^54^ *lncLINC00941* also has increased expression in several types of cancer and is associated with disease progression.^55^ Finally, *lncOIP5-AS1* is correlated with advanced clinical stages of cancer patients, worse prognosis and shorter overall survival.^56^ In BC, this lncRNA acts as a competitive endogenous RNA (ceRNA) promoting the expression of genes that favor progression and metastasis.^57^ Therefore, the alteration in the profile of these lncRNAs by **2** and **3** explains the inhibition of migration and the clonogenic capacity of MDA-MB-231 cells observed in the present study. In fact, the results of this study demonstrate that Cu­(II) complexes **2** and **3** act in a complex regulatory network associated with EMT. Furthermore, they suggest the potential of these complexes to overcome the limitations of conventional therapies including resistance mechanisms. Our findings open new perspectives for the development of treatment strategies based on Cu­(II) complexes that target lncRNA modulation for the management of TNBC.

## 5 Conclusion

Two novel Cu­(II) complexes, **2** and **3**, were active in TNBC cells, selectively reducing their viability and inhibiting proliferation, clonogenic capacity, and migration, with induction of apoptosis. These effects involved the modulation of lncRNAs *lncATB*, *lncBANCR*, *lncLINC00958*, *lncLINC00941*, and *lncOIP5-AS1*, molecules known to be involved in the regulation of invasion, migration, proliferation, and evasion of apoptosis (events currently evaluated). Therefore, this work not only confirms the efficacy of complexes **2** and **3** against TNBC cells but also mainly highlights the relevance of lncRNAs as strategic targets and the potential of Cu­(II) complexes as a therapeutic strategy that acts on post-transcriptional mechanisms that drive tumor aggressiveness.

## Supplementary Material





## Notes

Additional data on the X-ray crystallographic data obtained for the complex [Cu­(clmp)­(bta)­(CH_3_OH)]­NO_3_ (ZIP)
UV–vis spectra of copper complexes **1** and **4** at different concentrations (Figures S1–S4); infrared spectra of copper complexes and their free ligands (Figures S5–S8); mass spectra of copper complexes (Figures S9–S11); and cytotoxicity of complex ligands (Figure S12) (PDF)
