# Supplementary material for: Novel Copper(II) Complexes Containing β‑Diketones and Imines as Ligands Modulate the Expression of lncRNAs in Triple-Negative Breast Cancer Cells
Source: ACS Omega. 2025 Dec 19;11(1):687–99. doi: 10.1021/acsomega.5c06920 (PMC12809550; doi:10.1021/acsomega.5c06920)
Supplement: Supplementary file 1 [file ao5c06920_si_001.zip › mo_Lu_21_0m_tables.html]

mo\_Lu\_21\_0m


# mo\_Lu\_21\_0m

Table 1 Crystal data and structure refinement for mo\_Lu\_21\_0m.

| Identification code | mo\_Lu\_21\_0m |
| Empirical formula | C23H19ClCuF3N3O6 |
| Formula weight | 589.40 |
| Temperature/K | 293 |
| Crystal system | triclinic |
| Space group | P-1 |
| a/Å | 9.9145(4) |
| b/Å | 10.8443(4) |
| c/Å | 12.9702(5) |
| α/° | 72.431(2) |
| β/° | 67.921(2) |
| γ/° | 87.016(2) |
| Volume/Å3 | 1228.98(8) |
| Z | 2 |
| ρcalcg/cm3 | 1.593 |
| μ/mm‑1 | 1.065 |
| F(000) | 598.0 |
| Crystal size/mm3 | ? × ? × ? |
| Radiation | MoKα (λ = 0.71073) |
| 2Θ range for data collection/° | 4.436 to 52.894 |
| Index ranges | -12 ≤ h ≤ 12, -13 ≤ k ≤ 13, -16 ≤ l ≤ 16 |
| Reflections collected | 34584 |
| Independent reflections | 5052 [Rint = 0.0437, Rsigma = 0.0251] |
| Data/restraints/parameters | 5052/3/338 |
| Goodness-of-fit on F2 | 1.091 |
| Final R indexes [I>=2σ (I)] | R1 = 0.0394, wR2 = 0.0825 |
| Final R indexes [all data] | R1 = 0.0529, wR2 = 0.0913 |
| Largest diff. peak/hole / e Å-3 | 0.52/-0.57 |

Table 2 Fractional Atomic Coordinates (×104) and Equivalent Isotropic Displacement Parameters (Å2×103) for mo\_Lu\_21\_0m. Ueq is defined as 1/3 of the trace of the orthogonalised UIJ tensor.

| Atom | *x* | *y* | *z* | U(eq) |
| --- | --- | --- | --- | --- |
| Cu01 | 2240.1(3) | 7058.7(3) | 2418.9(3) | 36.81(10) |
| Cl1 | 5936.5(12) | 1619.0(9) | 4700.0(10) | 83.9(3) |
| F2 | -1436(2) | 9645.2(17) | 4931.3(14) | 59.6(5) |
| O2 | 1898(2) | 6172.6(17) | 4036.6(15) | 42.0(4) |
| O1 | 965(2) | 8388.6(17) | 2806.8(15) | 44.1(4) |
| N2 | 3230(2) | 5538.1(19) | 1940.9(17) | 33.6(4) |
| F1 | -67(3) | 10625.2(17) | 3191.3(18) | 85.6(7) |
| O32 | 8251(3) | 7144(3) | -888(3) | 91.9(9) |
| F3 | -1822(3) | 9319(2) | 3522(2) | 89.7(7) |
| O33 | 7406(4) | 8398(3) | 171(3) | 100.2(10) |
| O31 | 6006(3) | 7108(3) | 50(3) | 101.8(11) |
| N1 | 2177(2) | 7524(2) | 829.6(18) | 37.2(5) |
| O3 | 4302(2) | 8218(2) | 1913(2) | 60.2(6) |
| N3 | 7234(3) | 7564(2) | -235(2) | 51.2(6) |
| C22 | 4674(3) | 4941(3) | 3122(2) | 42.5(6) |
| C2 | 239(3) | 8391(2) | 3844(2) | 34.9(5) |
| C8 | 1158(3) | 3956(3) | 8144(3) | 55.9(8) |
| C11 | 1676(3) | 8578(3) | 289(2) | 47.8(7) |
| C10 | 501(3) | 5954(3) | 7088(2) | 40.0(6) |
| C18 | 3666(3) | 3249(2) | 2732(2) | 43.7(6) |
| C1 | -778(3) | 9504(3) | 3876(2) | 47.7(7) |
| C6 | 1647(3) | 4395(2) | 6110(3) | 43.2(6) |
| C3 | 226(3) | 7556(2) | 4875(2) | 37.6(5) |
| C4 | 1087(2) | 6468(2) | 4927(2) | 32.0(5) |
| C5 | 1052(2) | 5595(2) | 6067(2) | 33.3(5) |
| C9 | 557(3) | 5137(3) | 8115(2) | 49.7(7) |
| C21 | 5316(3) | 4040(3) | 3766(3) | 47.6(7) |
| C7 | 1696(3) | 3581(3) | 7147(3) | 54.9(8) |
| C14 | 2577(3) | 6677(3) | -748(2) | 49.4(7) |
| C20 | 5113(3) | 2746(3) | 3892(3) | 47.9(7) |
| C17 | 3859(2) | 4552(2) | 2599(2) | 33.5(5) |
| C19 | 4302(3) | 2341(3) | 3382(3) | 51.2(7) |
| C15 | 2631(3) | 6586(2) | 314(2) | 37.2(5) |
| C16 | 3216(3) | 5498(2) | 971(2) | 37.3(5) |
| C12 | 1586(4) | 8724(3) | -778(3) | 58.6(8) |
| C13 | 2027(4) | 7764(3) | -1296(3) | 59.1(8) |
| C31 | 4413(5) | 9480(4) | 1957(5) | 104.7(16) |

Table 3 Anisotropic Displacement Parameters (Å2×103) for mo\_Lu\_21\_0m. The Anisotropic displacement factor exponent takes the form: -2π2[h2a\*2U11+2hka\*b\*U12+…].

| Atom | U11 | U22 | U33 | U23 | U13 | U12 |
| --- | --- | --- | --- | --- | --- | --- |
| Cu01 | 40.65(18) | 38.10(17) | 35.01(17) | -17.07(13) | -14.62(13) | 12.87(13) |
| Cl1 | 87.2(7) | 56.6(5) | 103.2(7) | 1.7(5) | -52.9(6) | 18.4(5) |
| F2 | 69.7(11) | 60.0(10) | 51.0(10) | -28.6(8) | -19.7(9) | 35.3(9) |
| O2 | 48.1(10) | 43.7(10) | 36.9(9) | -17.1(8) | -17.6(8) | 22.1(8) |
| O1 | 54.2(11) | 41.6(10) | 34.3(9) | -13.2(8) | -15.0(8) | 18.8(8) |
| N2 | 27.1(10) | 34.8(11) | 39.1(11) | -15.6(9) | -9.7(8) | 4.9(8) |
| F1 | 119.6(18) | 37.9(10) | 66.3(12) | -6.3(9) | -9.5(12) | 25.4(11) |
| O32 | 57.7(16) | 118(2) | 100(2) | -59.1(19) | -10.1(15) | 13.2(15) |
| F3 | 98.3(16) | 105.8(17) | 123.5(19) | -73.2(15) | -83.5(15) | 67.5(14) |
| O33 | 146(3) | 64.6(17) | 134(3) | -55.2(18) | -84(2) | 25.1(17) |
| O31 | 52.8(15) | 117(2) | 129(3) | -74(2) | 4.6(16) | -14.9(15) |
| N1 | 34.0(11) | 41.4(12) | 35.0(11) | -13.6(9) | -10.7(9) | 6.2(9) |
| O3 | 49.7(12) | 58.9(13) | 70.1(15) | -29.8(11) | -11.6(11) | -5.8(10) |
| N3 | 59.3(16) | 44.2(13) | 49.8(14) | -16.0(11) | -19.3(12) | 8.1(12) |
| C22 | 38.1(14) | 34.0(13) | 62.2(17) | -19.9(12) | -23.0(13) | 8.4(11) |
| C2 | 35.7(13) | 33.8(12) | 40.1(13) | -16.5(10) | -16.6(11) | 9.3(10) |
| C8 | 56.1(18) | 54.7(18) | 49.4(17) | 7.7(14) | -28.1(15) | -10.8(14) |
| C11 | 52.2(17) | 46.7(16) | 43.8(15) | -14.8(12) | -17.6(13) | 11.6(13) |
| C10 | 39.2(14) | 42.4(14) | 39.4(13) | -12.2(11) | -16.2(11) | 2.5(11) |
| C18 | 40.9(14) | 37.7(14) | 53.1(16) | -18.9(12) | -14.2(12) | 0.3(11) |
| C1 | 62.1(18) | 43.1(15) | 44.6(15) | -20.0(13) | -25.0(14) | 24.9(13) |
| C6 | 47.5(15) | 33.7(13) | 53.3(16) | -13.2(12) | -24.5(13) | 3.9(11) |
| C3 | 43.0(14) | 37.3(13) | 34.9(12) | -15.7(10) | -15.0(11) | 13.9(11) |
| C4 | 30.9(12) | 32.6(12) | 38.2(12) | -14.9(10) | -16.5(10) | 4.7(9) |
| C5 | 30.0(12) | 32.1(12) | 39.4(13) | -8.4(10) | -16.3(10) | -1.0(9) |
| C9 | 48.6(16) | 59.9(18) | 36.5(14) | -6.3(13) | -17.0(12) | -6.1(14) |
| C21 | 39.1(14) | 48.2(16) | 62.6(18) | -19.5(14) | -25.9(13) | 9.6(12) |
| C7 | 61.7(19) | 33.0(14) | 70(2) | -1.5(14) | -35.8(17) | 2.3(13) |
| C14 | 48.3(16) | 63.8(19) | 40.7(15) | -26.0(14) | -14.1(13) | 5.3(14) |
| C20 | 40.4(15) | 40.4(15) | 53.8(16) | -6.8(12) | -15.0(13) | 10.2(12) |
| C17 | 26.2(11) | 34.5(12) | 38.7(13) | -15.6(10) | -8.1(10) | 7.6(9) |
| C19 | 53.4(17) | 31.8(14) | 62.4(18) | -12.6(13) | -16.6(15) | 0.9(12) |
| C15 | 28.4(12) | 44.5(14) | 36.8(13) | -16.0(11) | -7.1(10) | 0.5(10) |
| C16 | 28.6(12) | 41.9(14) | 42.6(14) | -21.3(11) | -8.1(10) | 3.0(10) |
| C12 | 66(2) | 63(2) | 46.4(16) | -10.6(15) | -27.1(15) | 15.5(16) |
| C13 | 64(2) | 80(2) | 37.4(15) | -17.8(15) | -23.8(14) | 10.3(17) |
| C31 | 100(3) | 69(3) | 140(4) | -46(3) | -26(3) | -20(2) |

Table 4 Bond Lengths for mo\_Lu\_21\_0m.

| Atom | Atom | Length/Å |  | Atom | Atom | Length/Å |
| --- | --- | --- | --- | --- | --- | --- |
| Cu01 | O2 | 1.9294(17) |  | C22 | C17 | 1.382(3) |
| Cu01 | O1 | 1.9241(17) |  | C2 | C1 | 1.532(3) |
| Cu01 | N2 | 2.0146(19) |  | C2 | C3 | 1.366(3) |
| Cu01 | N1 | 1.993(2) |  | C8 | C9 | 1.380(4) |
| Cu01 | O3 | 2.225(2) |  | C8 | C7 | 1.378(5) |
| Cl1 | C20 | 1.739(3) |  | C11 | C12 | 1.381(4) |
| F2 | C1 | 1.330(3) |  | C10 | C5 | 1.396(3) |
| O2 | C4 | 1.257(3) |  | C10 | C9 | 1.379(4) |
| O1 | C2 | 1.266(3) |  | C18 | C17 | 1.386(3) |
| N2 | C17 | 1.428(3) |  | C18 | C19 | 1.383(4) |
| N2 | C16 | 1.278(3) |  | C6 | C5 | 1.395(3) |
| F1 | C1 | 1.316(3) |  | C6 | C7 | 1.384(4) |
| O32 | N3 | 1.218(3) |  | C3 | C4 | 1.420(3) |
| F3 | C1 | 1.326(4) |  | C4 | C5 | 1.485(3) |
| O33 | N3 | 1.223(3) |  | C21 | C20 | 1.380(4) |
| O31 | N3 | 1.216(3) |  | C14 | C15 | 1.373(4) |
| N1 | C11 | 1.328(3) |  | C14 | C13 | 1.382(4) |
| N1 | C15 | 1.350(3) |  | C20 | C19 | 1.374(4) |
| O3 | C31 | 1.399(4) |  | C15 | C16 | 1.465(4) |
| C22 | C21 | 1.378(4) |  | C12 | C13 | 1.370(4) |

Table 5 Bond Angles for mo\_Lu\_21\_0m.

| Atom | Atom | Atom | Angle/˚ |  | Atom | Atom | Atom | Angle/˚ |
| --- | --- | --- | --- | --- | --- | --- | --- | --- |
| O2 | Cu01 | N2 | 91.19(8) |  | F1 | C1 | F2 | 106.0(2) |
| O2 | Cu01 | N1 | 162.30(8) |  | F1 | C1 | F3 | 107.7(3) |
| O2 | Cu01 | O3 | 99.67(9) |  | F1 | C1 | C2 | 111.5(2) |
| O1 | Cu01 | O2 | 92.43(7) |  | F3 | C1 | F2 | 106.8(2) |
| O1 | Cu01 | N2 | 167.85(8) |  | F3 | C1 | C2 | 110.7(2) |
| O1 | Cu01 | N1 | 91.80(8) |  | C7 | C6 | C5 | 120.4(3) |
| O1 | Cu01 | O3 | 95.75(8) |  | C2 | C3 | C4 | 122.5(2) |
| N2 | Cu01 | O3 | 95.07(8) |  | O2 | C4 | C3 | 123.2(2) |
| N1 | Cu01 | N2 | 81.40(8) |  | O2 | C4 | C5 | 116.2(2) |
| N1 | Cu01 | O3 | 97.00(9) |  | C3 | C4 | C5 | 120.6(2) |
| C4 | O2 | Cu01 | 128.47(15) |  | C10 | C5 | C4 | 122.3(2) |
| C2 | O1 | Cu01 | 123.58(16) |  | C6 | C5 | C10 | 118.8(2) |
| C17 | N2 | Cu01 | 125.78(15) |  | C6 | C5 | C4 | 118.8(2) |
| C16 | N2 | Cu01 | 113.09(17) |  | C10 | C9 | C8 | 120.3(3) |
| C16 | N2 | C17 | 121.1(2) |  | C22 | C21 | C20 | 118.9(3) |
| C11 | N1 | Cu01 | 127.78(18) |  | C8 | C7 | C6 | 120.0(3) |
| C11 | N1 | C15 | 119.1(2) |  | C15 | C14 | C13 | 118.4(3) |
| C15 | N1 | Cu01 | 112.94(17) |  | C21 | C20 | Cl1 | 118.4(2) |
| C31 | O3 | Cu01 | 126.0(2) |  | C19 | C20 | Cl1 | 120.0(2) |
| O32 | N3 | O33 | 122.5(3) |  | C19 | C20 | C21 | 121.5(3) |
| O31 | N3 | O32 | 118.6(3) |  | C22 | C17 | N2 | 117.4(2) |
| O31 | N3 | O33 | 118.8(3) |  | C22 | C17 | C18 | 120.1(2) |
| C21 | C22 | C17 | 120.4(2) |  | C18 | C17 | N2 | 122.5(2) |
| O1 | C2 | C1 | 111.4(2) |  | C20 | C19 | C18 | 119.4(3) |
| O1 | C2 | C3 | 129.7(2) |  | N1 | C15 | C14 | 122.1(2) |
| C3 | C2 | C1 | 118.9(2) |  | N1 | C15 | C16 | 114.2(2) |
| C7 | C8 | C9 | 120.1(3) |  | C14 | C15 | C16 | 123.7(2) |
| N1 | C11 | C12 | 121.5(3) |  | N2 | C16 | C15 | 117.7(2) |
| C9 | C10 | C5 | 120.3(3) |  | C13 | C12 | C11 | 119.4(3) |
| C19 | C18 | C17 | 119.7(3) |  | C12 | C13 | C14 | 119.3(3) |
| F2 | C1 | C2 | 113.8(2) |  |  |  |  |  |

Table 6 Torsion Angles for mo\_Lu\_21\_0m.

| A | B | C | D | Angle/˚ |  | A | B | C | D | Angle/˚ |
| --- | --- | --- | --- | --- | --- | --- | --- | --- | --- | --- |
| Cu01 | O2 | C4 | C3 | 1.2(4) |  | C3 | C2 | C1 | F1 | 129.0(3) |
| Cu01 | O2 | C4 | C5 | -179.67(15) |  | C3 | C2 | C1 | F3 | -111.1(3) |
| Cu01 | O1 | C2 | C1 | -174.82(17) |  | C3 | C4 | C5 | C10 | -16.7(3) |
| Cu01 | O1 | C2 | C3 | 3.4(4) |  | C3 | C4 | C5 | C6 | 166.0(2) |
| Cu01 | N2 | C17 | C22 | -42.7(3) |  | C5 | C10 | C9 | C8 | 0.0(4) |
| Cu01 | N2 | C17 | C18 | 136.7(2) |  | C5 | C6 | C7 | C8 | -0.2(4) |
| Cu01 | N2 | C16 | C15 | 5.6(3) |  | C9 | C8 | C7 | C6 | 0.6(4) |
| Cu01 | N1 | C11 | C12 | -174.4(2) |  | C9 | C10 | C5 | C6 | 0.4(4) |
| Cu01 | N1 | C15 | C14 | 175.4(2) |  | C9 | C10 | C5 | C4 | -176.8(2) |
| Cu01 | N1 | C15 | C16 | -6.2(3) |  | C21 | C22 | C17 | N2 | -179.8(2) |
| Cl1 | C20 | C19 | C18 | -179.3(2) |  | C21 | C22 | C17 | C18 | 0.8(4) |
| O2 | C4 | C5 | C10 | 164.1(2) |  | C21 | C20 | C19 | C18 | -0.4(4) |
| O2 | C4 | C5 | C6 | -13.2(3) |  | C7 | C8 | C9 | C10 | -0.6(4) |
| O1 | C2 | C1 | F2 | -172.4(2) |  | C7 | C6 | C5 | C10 | -0.4(4) |
| O1 | C2 | C1 | F1 | -52.6(3) |  | C7 | C6 | C5 | C4 | 177.0(2) |
| O1 | C2 | C1 | F3 | 67.3(3) |  | C14 | C15 | C16 | N2 | 178.7(2) |
| O1 | C2 | C3 | C4 | -0.8(4) |  | C17 | N2 | C16 | C15 | -176.4(2) |
| N1 | C11 | C12 | C13 | -0.3(5) |  | C17 | C22 | C21 | C20 | -0.9(4) |
| N1 | C15 | C16 | N2 | 0.4(3) |  | C17 | C18 | C19 | C20 | 0.3(4) |
| C22 | C21 | C20 | Cl1 | 179.6(2) |  | C19 | C18 | C17 | N2 | -179.9(2) |
| C22 | C21 | C20 | C19 | 0.7(4) |  | C19 | C18 | C17 | C22 | -0.5(4) |
| C2 | C3 | C4 | O2 | -1.7(4) |  | C15 | N1 | C11 | C12 | 1.1(4) |
| C2 | C3 | C4 | C5 | 179.2(2) |  | C15 | C14 | C13 | C12 | 1.4(5) |
| C11 | N1 | C15 | C14 | -0.7(4) |  | C16 | N2 | C17 | C22 | 139.5(2) |
| C11 | N1 | C15 | C16 | 177.6(2) |  | C16 | N2 | C17 | C18 | -41.1(3) |
| C11 | C12 | C13 | C14 | -1.0(5) |  | C13 | C14 | C15 | N1 | -0.5(4) |
| C1 | C2 | C3 | C4 | 177.3(2) |  | C13 | C14 | C15 | C16 | -178.7(3) |
| C3 | C2 | C1 | F2 | 9.2(4) |  |  |  |  |  |  |

Table 7 Hydrogen Atom Coordinates (Å×104) and Isotropic Displacement Parameters (Å2×103) for mo\_Lu\_21\_0m.

| Atom | *x* | *y* | *z* | U(eq) |
| --- | --- | --- | --- | --- |
| H3 | 5083(18) | 8110(20) | 1370(20) | 90 |
| H22 | 4789.96 | 5816.21 | 3038.64 | 51 |
| H8 | 1200.54 | 3412.21 | 8837.94 | 67 |
| H11 | 1379.04 | 9232.09 | 635.95 | 57 |
| H10 | 95.15 | 6748.76 | 7074.8 | 48 |
| H18 | 3111.24 | 2985.69 | 2385.72 | 52 |
| H6 | 2013.72 | 4139.4 | 5438.4 | 52 |
| H3A | -370.05 | 7705.02 | 5569.51 | 45 |
| H9 | 187.75 | 5382.48 | 8791.7 | 60 |
| H21 | 5876.31 | 4300.57 | 4109.43 | 57 |
| H7 | 2091.65 | 2781.36 | 7170.45 | 66 |
| H14 | 2902.33 | 6022.4 | -1091.37 | 59 |
| H19 | 4182.05 | 1464.5 | 3473.61 | 61 |
| H16 | 3564.07 | 4802.22 | 690.68 | 45 |
| H12 | 1227.72 | 9466.81 | -1142.45 | 70 |
| H13 | 1958.17 | 7843.82 | -2008.02 | 71 |
| H31A | 4395.44 | 10088.99 | 1250.44 | 157 |
| H31B | 5312.4 | 9621.47 | 2035.81 | 157 |
| H31C | 3608.13 | 9594.47 | 2613.91 | 157 |

Experimental

Single crystals of C23H19ClCuF3N3O6
[mo\_Lu\_21\_0m]
were
[].
A suitable crystal was selected and
[]
on a
Bruker D8 Venture
diffractometer. The crystal was kept at 293 K during data collection.
Using Olex2 [1], the structure was solved with the
SHELXT
[2] structure solution program using
Intrinsic Phasing
and refined with the
SHELXL
[3] refinement package using
Least Squares
minimisation.

1. Dolomanov, O.V., Bourhis, L.J., Gildea, R.J, Howard, J.A.K. & Puschmann, H.
   (2009), J. Appl. Cryst. 42, 339-341.
2. Sheldrick, G.M. (2015). Acta Cryst. A71, 3-8.
3. Sheldrick, G.M. (2015). Acta Cryst. C71, 3-8.

Crystal structure determination of
[mo\_Lu\_21\_0m]

**Crystal Data**
for C23H19ClCuF3N3O6 (*M*=589.40 g/mol):
triclinic, space group P-1 (no. 2),
*a* = 9.9145(4) Å, *b* = 10.8443(4) Å, *c* = 12.9702(5) Å, *α* = 72.431(2)°, *β* = 67.921(2)°, *γ* = 87.016(2)°,
*V*= 1228.98(8) Å3,
*Z* = 2,
*T* = 293 K,
μ(MoKα) = 1.065 mm-1,
*Dcalc* = 1.593 g/cm3,
34584 reflections measured (4.436° ≤ 2Θ ≤ 52.894°),
5052 unique (*R*int = 0.0437, Rsigma = 0.0251) which were used in all calculations.
The final *R*1 was 0.0394
(I > 2σ(I)) and *wR*2 was 0.0913 (all data).

Refinement model description

Number of restraints - 3,
number of constraints - unknown.

Details:

```
1. Fixed Uiso
```

This report has been created with Olex2, compiled on
2024.02.16 svn.r378c4104 for OlexSys. Please
let us know
if there are any errors or if you would like to have additional features.
