# Supplementary material for: Novel Copper(II) Complexes Containing β‑Diketones and Imines as Ligands Modulate the Expression of lncRNAs in Triple-Negative Breast Cancer Cells
Source: ACS Omega. 2025 Dec 19;11(1):687–99. doi: 10.1021/acsomega.5c06920 (PMC12809550; doi:10.1021/acsomega.5c06920)
Supplement: Supplementary file 1 [file ao5c06920_si_001.zip › simple text.docx]

Guilherme Pereira Guedes^a^, Jackson A. L. C. Resende,

a Universidade Federal Fluminense, Instituto de Química, Outeiro S. João Batista S/N, 24020-141, Niterói, RJ, Brazil

b Universidade Federal de Mato Grosso, Instituto de Ciências Exatas e da Terra, Av. Valdon Varjão, 6390, 78600-000, Barra do Garças, MT, Brazil

The compound crystallizes as a nitrate salt, with the copper atom exhibiting square planar coordination with axial distortion. A weakly coordinated ethanol molecule is observed at a long intermolecular distance (2.225(2) Å), which easily dissociates from the metal in solution. The ethanol molecule plays a role in the crystal packing of the compound, connecting the complex cation to the nitrate anion in the structure.


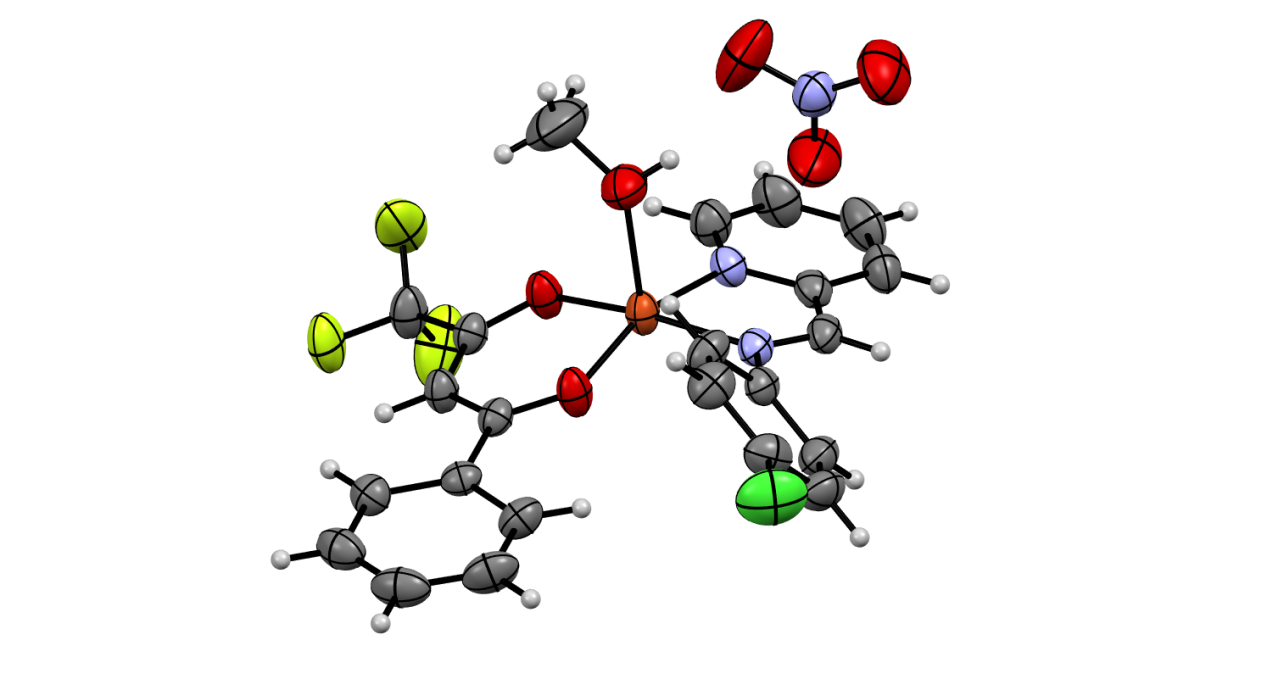


 X-ray crystal structure of the compound

Experimental:

X-ray diffraction data was carried out in Bruker D8 Venture with radiation MoKα (λ = 0.71073 Å). The structure was solved by Intrisinc Phasing and refined by full-matrix least squares on F2 with SHELX package [1]. The positions of hydrogen atoms were generated geometrically and refined according to a riding model. All non-hydrogen atoms were refined anisotropically. Crystallographic data for compound X: Crystal Data for CuC_23_H_19_ClF_3_N_3_O_6_ (M =589.40 g/mol): triclinic, space group P-1 (no. 2), a = 9.9145(4) Å, b = 10.8443(4) Å, c = 12.9702(5) Å, α = 72.431(2)°, β = 67.921(2)°, γ = 87.016(2)°, V = 1228.98(8) Å^3^, Z = 2, T = 293 K, μ(MoKα) = 1.065 mm^-1^, Dcalc = 1.593 g/cm^3^, 34584 reflections measured (4.436° ≤ 2Θ ≤ 52.894°), 5052 unique (Rint = 0.0437, Rsigma = 0.0251) which were used in all calculations. The final R_1_ was 0.0394 (I > 2σ(I)) and wR2 was 0.0913 (all data). Crystallographic data has been deposited with the Cambridge Crystallographic Data Centre database number 2372590.

[1] Sheldrick, G. M. (2015). Crystal structure refinement with SHELXL. Acta Cryst. C71, 3-8.
