# Supplementary figures and images for: Novel Copper(II) Complexes Containing β‑Diketones and Imines as Ligands Modulate the Expression of lncRNAs in Triple-Negative Breast Cancer Cells
Source: ACS Omega. 2025 Dec 19;11(1):687–99. doi: 10.1021/acsomega.5c06920 (PMC12809550; doi:10.1021/acsomega.5c06920)

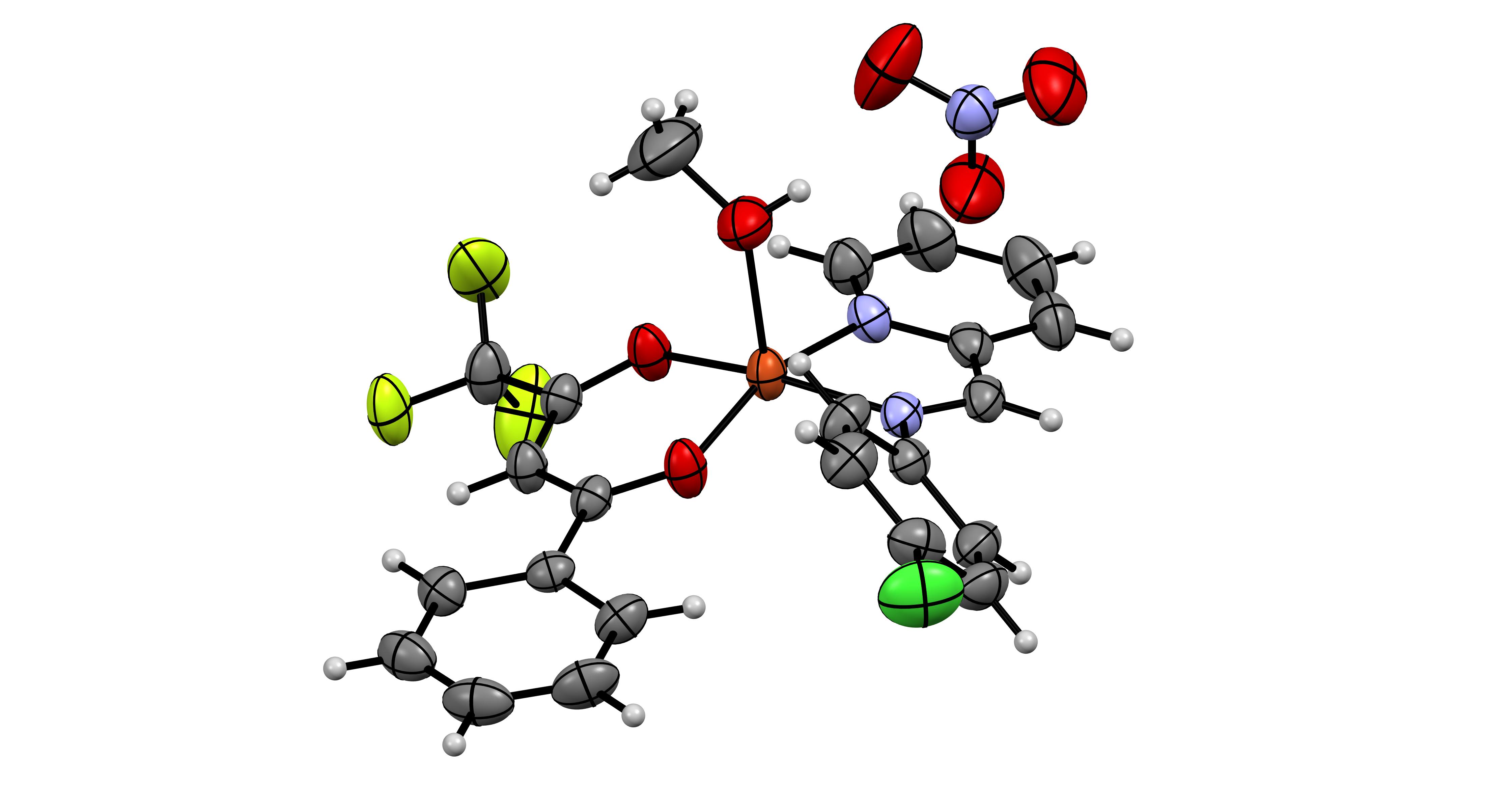

Supplement: Supplementary file 1 [file ao5c06920_si_001.zip › lu_21.jpg]
