# Supplementary material for: Novel Copper(II) Complexes Containing β‑Diketones and Imines as Ligands Modulate the Expression of lncRNAs in Triple-Negative Breast Cancer Cells
Source: ACS Omega. 2025 Dec 19;11(1):687–99. doi: 10.1021/acsomega.5c06920 (PMC12809550; doi:10.1021/acsomega.5c06920)

Supporting Information

Title: Novel copper(II) complexes containing β-diketones and imines as ligands modulate the expression of lncRNAs in triple-negative breast cancer cells

Authorship

Gislaine Gonçalves Rocha[1≠], Luana Munique Sousa Ramos[2≠], Laryssa Aparecida Sales Barbosa[1], Raoni Pais Siqueira[1], Fernanda Cardoso da Silva[1], Douglas Cardoso Brandão[1], Paula Marynella Alves Pereira Lima[1], André Carlos Pereira de Matos[1], André Luiz Bogado[3], Guilherme Pereira Guedes[4], Jackson Antonio Lamounier Camargo Resende[5], Gabriele de Menezes Pereira[6], Pedro Paulo Corbi[6], Wendell Guerra[2*], Thaise Gonçalves de Araújo[1,7*]

Affiliation

[1]Laboratory of Genetics and Biotechnology, Institute of Biotechnology, Universidade Federal de Uberlândia, Patos de Minas, MG 38700-002, Brazil; gislaineg.rocha08@gmail.com (G.G.R.); laryssaasales@gmail.com (L.A.S.B.); raoni.siqueira@ufu.br (R.P.S.); fernanda.cardoso95@yahoo.com (F.C.S.); douglascb@unipam.edu.br (D.C.B.); paulamarynella@hotmail.com (P.M.A.P.L.); andre3ptm@gmail.com (A.C.P.M.); tgaraujo@ufu.br (T.G.A.).

[2]Institute of Chemistry, Universidade Federal de Uberlândia, Av. João Naves de Ávila - 2121 - Bloco 1D - Bairro Santa Mônica, Uberlândia, MG, CEP 38400-902, Brazil; luana_munique@hotmail.com (L.M.S.R); wendell.guerra@ufu.br (W.G).

[3]Institute of Exact and Natural Sciences of Pontal, Universidade Federal de Uberlândia, Ituiutaba, MG 38304-402, Brazil; bogado@ufu.br (A.L.B.).

[4]Institute of Chemistry, Universidade Federal Fluminense, Niterói, RJ 24020-141, Brazil; guilherme_guedes@id.uff.br (G.P.G).

[5]Institute of Exact and Earth Sciences, Universidade Federal de Mato Grosso, Barra do Garças, MT 78600-000, Brazil; jackson.resende@ufmt.br (J.A.L.C.R).

[6]Institute of Chemistry, Universidade Estadual de Campinas, Campinas, SP 13083-970, Brazil; gabriele_menezzes@hotmail.com (G.M.P.); ppcorbi@unicamp.br (P.P.C).

[7]Laboratory of Nanobiotechnology Prof. Dr. Luiz Ricardo Goulart Filho, Institute of Biotechnoloy, Universidade Federal de Uberlândia, Uberlandia-MG, 38405-302, Brazil; tgaraujo@ufu.br (T.G.A.).

Figure S1. UV-Vis spectrum of complex **1** in acetonitrile (1.0×10^{-5} M).

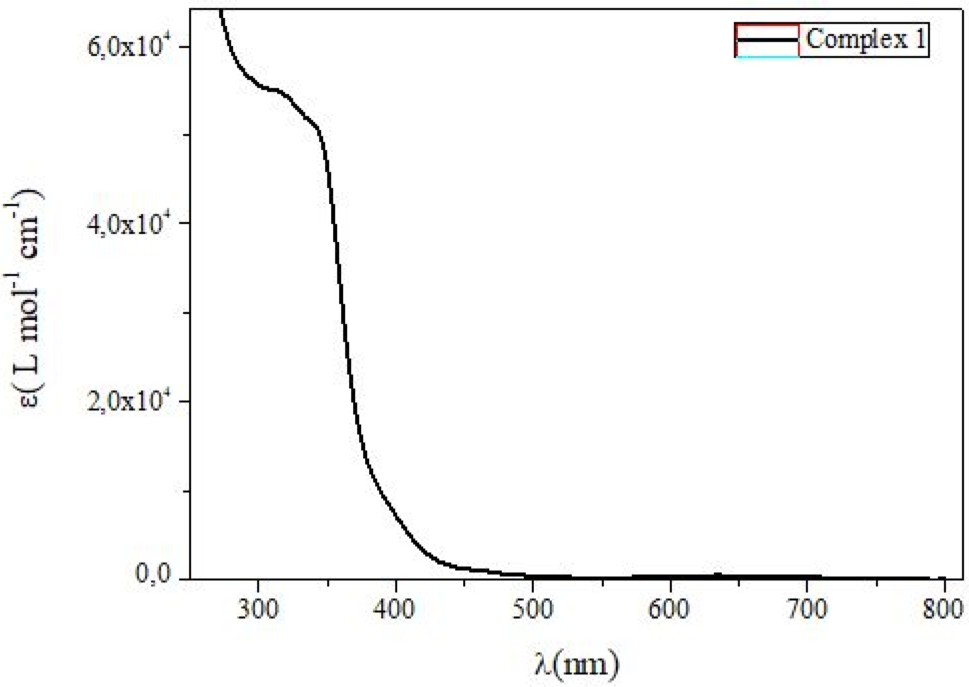

Figure S2. UV-Vis spectrum of complex **1** in acetonitrile (1.0×10^{-3} M).

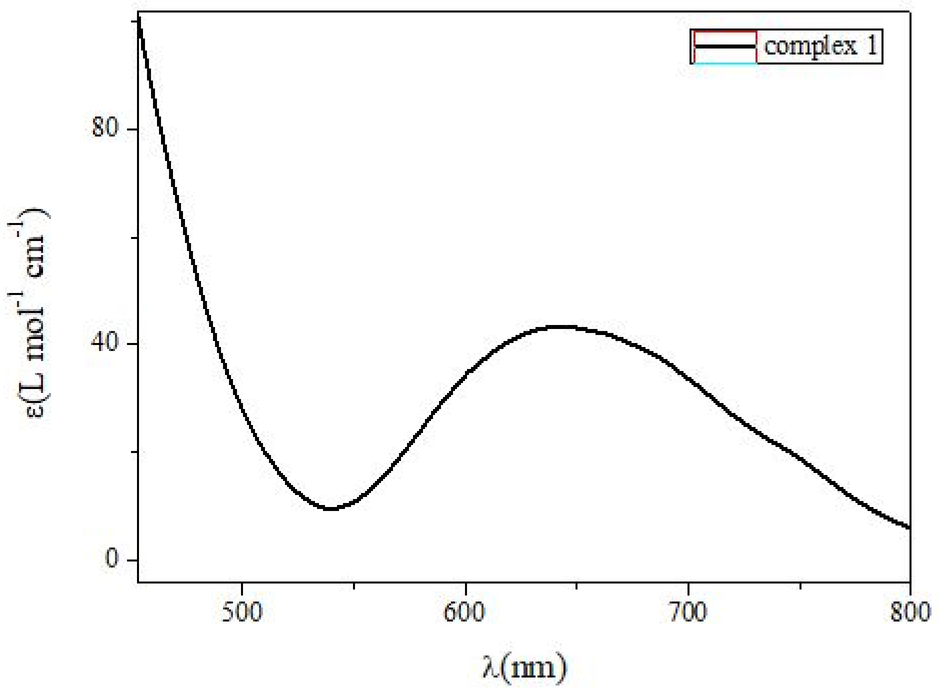

Figure S3. UV-Vis spectrum of complex **4** in acetonitrile (1.0×10^{-5} M).

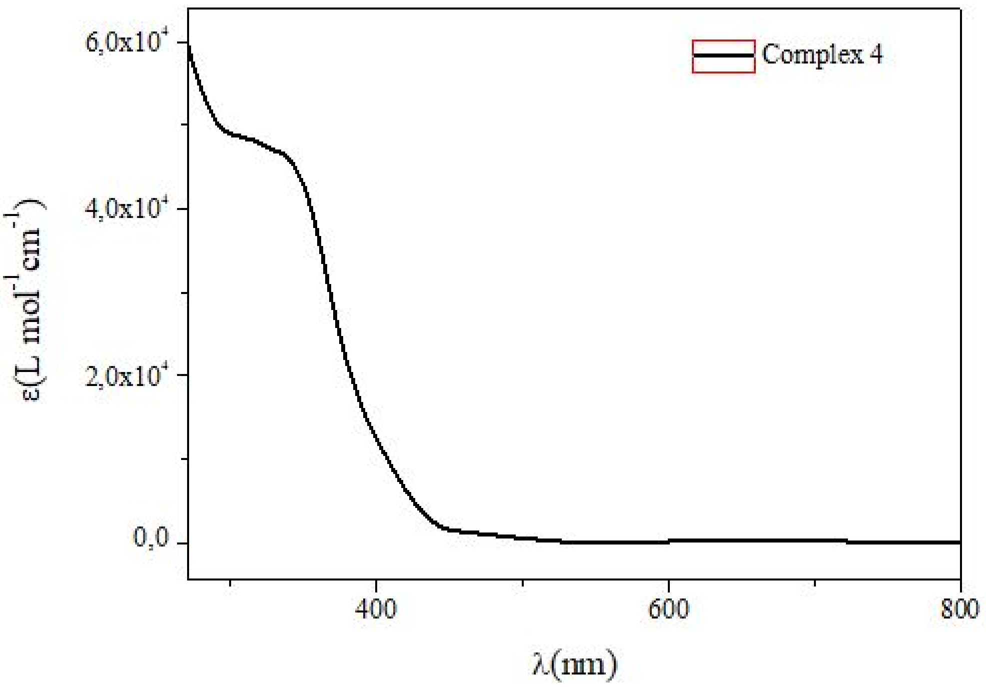

Figure S4. UV-Vis spectrum of complex **4** in acetonitrile (1.0×10^{-3} M).

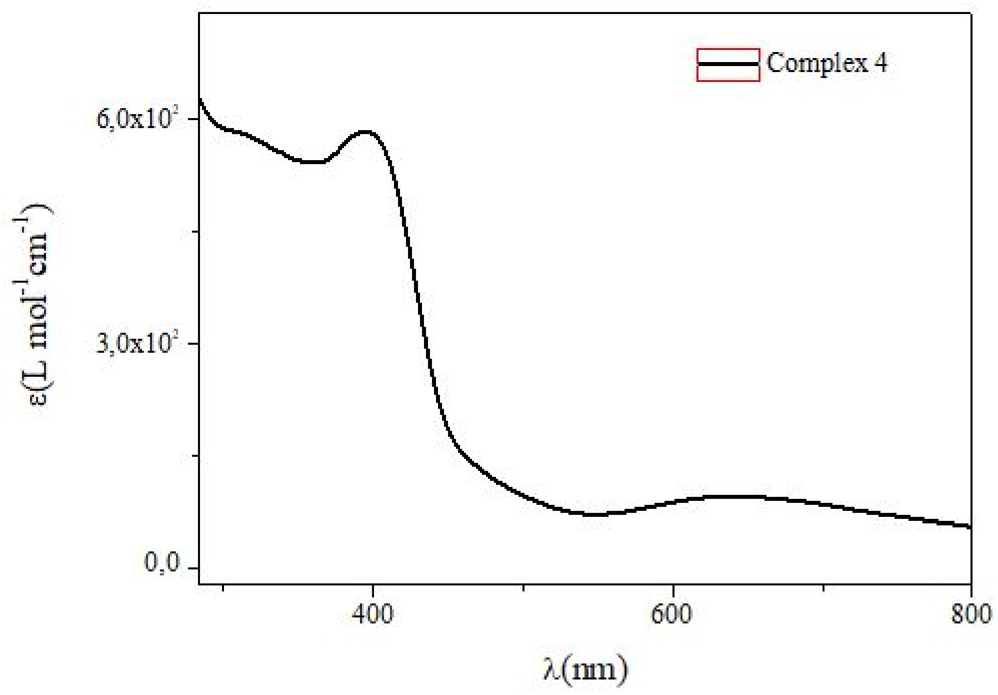

Figure S5. FTIR spectra of complex **1** and its free ligands.

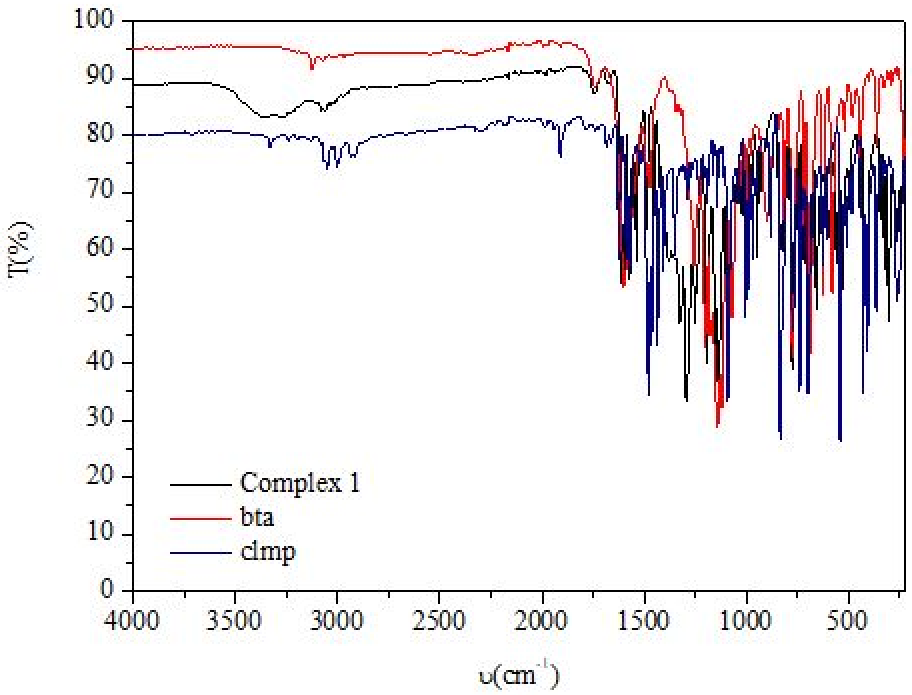

Figure S6. FTIR spectra of complex **2** and its free ligands.

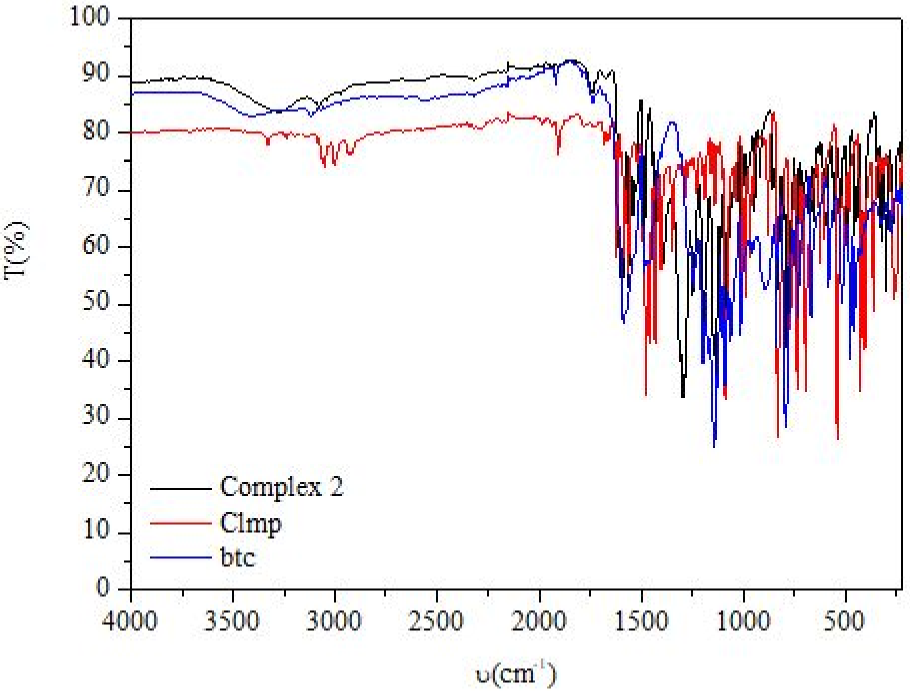

Figure S7. FTIR spectra of complex **3** and its free ligands.

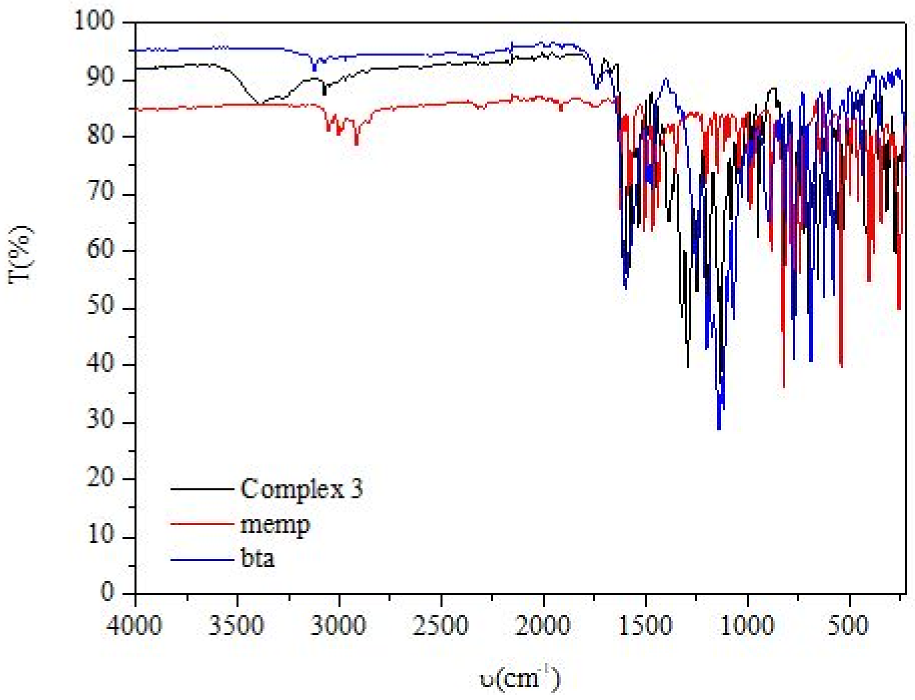

Figure S8. FTIR spectra of complex **4** and its free ligands.

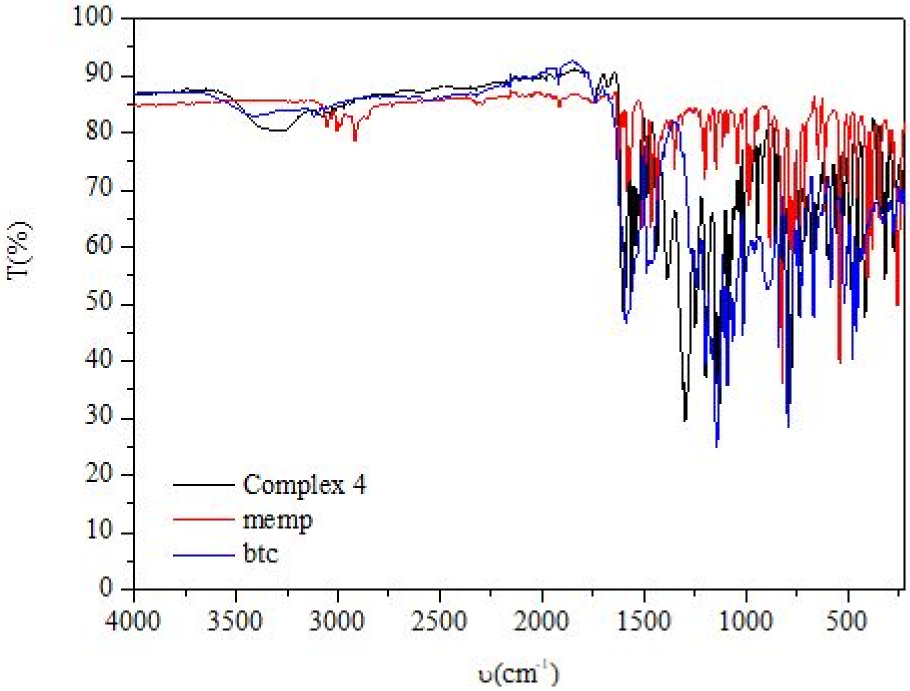

Figure S9. Mass spectrum of complex **1**. The charged complex ion observed was $[M - NO_3]^+$.

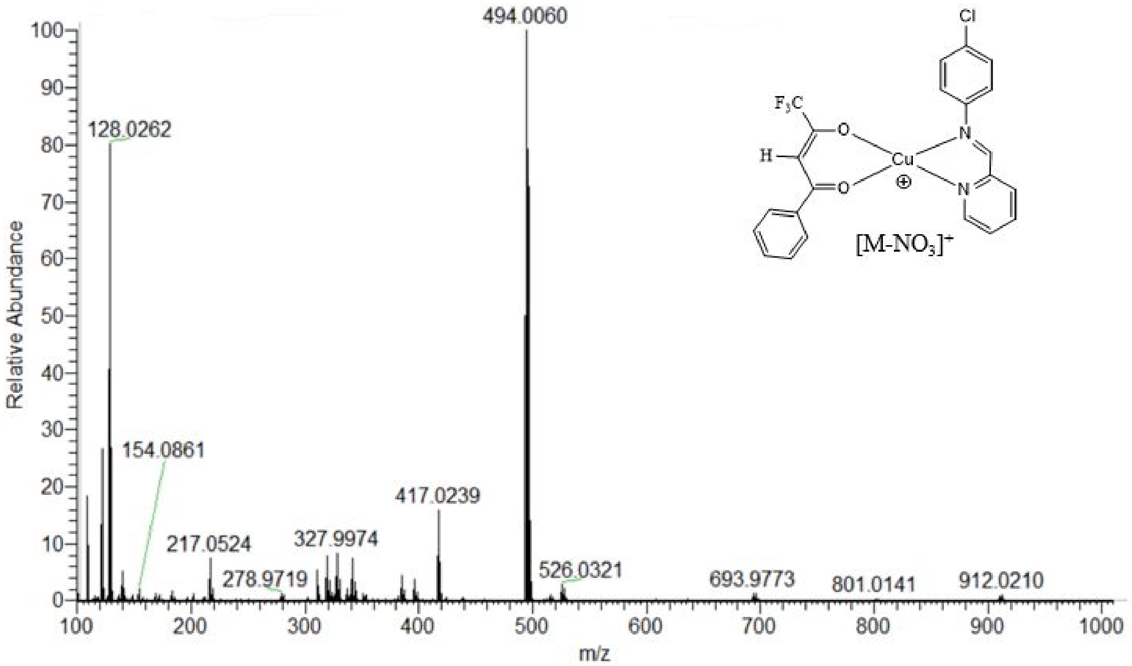

Figure S10. Mass spectrum of complex **2**. The charged complex ion observed was [M – $NO_3]^+$.

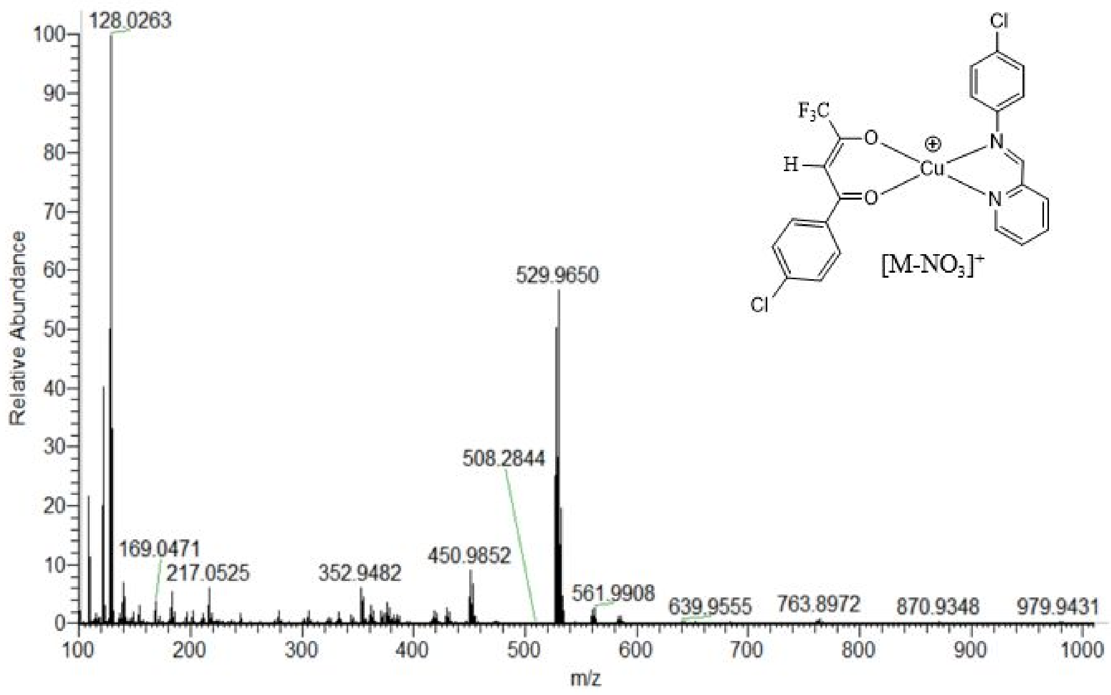

Figure S11. Mass spectrum of complex **3**. The charged complex ion observed was [M – NO_3]$^+$.

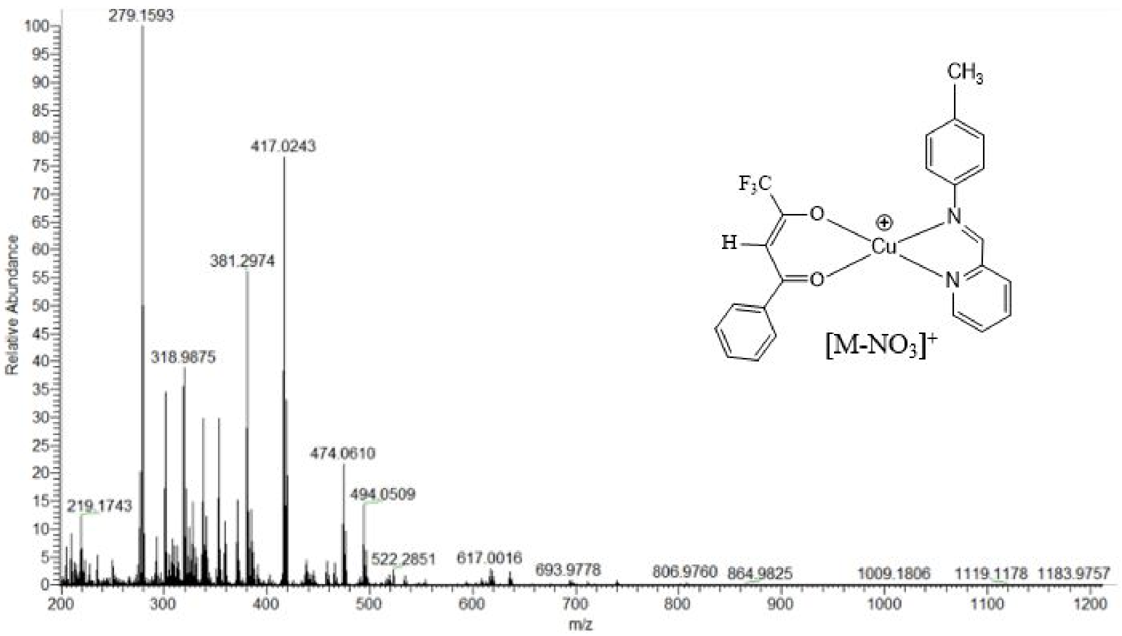
279.1593
417.0243
381.2974
318.9875
474.0610
494.0509
219.1743
522.2851
617.0016
693.9778
806.9760
864.9825
1009.1806
1119.1178
1183.9757
Relative Abundance
m/z
F_3C
O
H
Cu
N
N
O
CH_3
$[M\text{-}NO_3]^+$

Figure S12. Viability of MCF-10A and MDA-MB-231 cell lines after treatment with BTACl, Clmp, BTA, and Memp ligands.

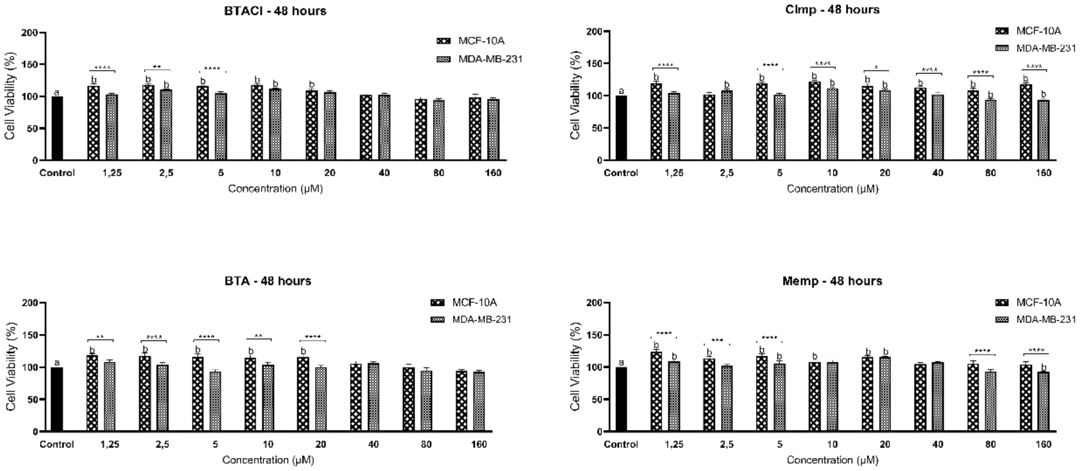

Supplement: Supplementary file 2 [file ao5c06920_si_002.pdf]
